# Thyroid doses estimated for a cohort of people exposed to fallout from atmospheric nuclear weapons testing at the semipalatinsk nuclear test site, Kazakhstan

**DOI:** 10.1007/s00411-025-01155-x

**Published:** 2025-11-17

**Authors:** Vladimir Drozdovitch, Alexandra Lipikhina, Kazbek Apsalikov, Yulia Brait, Alik Tokanov, Gani Yessilkanov, Rafail Rosenson, André Bouville, Evgenia Ostroumova

**Affiliations:** 1 Division of Cancer Epidemiology and Genetics, National Cancer Institute, NIH, DHHS, Bethesda, MD USA; 2 Scientific Research Institute of Radiation Medicine and Ecology, NCJSC Semey Medical University, Semey, Republic of Kazakhstan; 3 NCJSC Astana Medical University, Astana, Republic of Kazakhstan; 4 National Cancer Institute, NIH, DHHS, Bethesda, MD USA; 5 Environment and Lifestyle Epidemiology Branch, International Agency for Research on Cancer (IARC/WHO), Lyon, France

**Keywords:** Atmospheric nuclear weapons testing, Dose reconstruction, Thyroid, Semipalatinsk, Kazakhstan

## Abstract

Thyroid doses were estimated for a cohort of 3,183 individuals who were exposed to fallout from atmospheric nuclear weapons tests conducted at the Semipalatinsk Nuclear Test Site (SNTS), Kazakhstan, between 1949 and 1962. The study participants were mostly younger than 21 years of age at the time of their first exposure and lived in settlements near the SNTS. Individual thyroid doses from external irradiation from gamma-emitting radionuclides deposited on the ground as well as internal irradiation from intake of ^131^I and short-lived radiotellurium and radioiodine isotopes (^132^Te+^132^I, ^133^I, and ^135^I) with locally produced foodstuffs and inhalation of contaminated air during the passage of the radioactive cloud were reconstructed for the cohort. Estimated thyroid doses from external irradiation ranged from 4.9 × 10^−5^ Gy to 0.58 Gy (arithmetic mean (AM) dose was 0.048 Gy, median dose was 0.023 Gy), internal thyroid doses from ingestion of ^131^I, ^132^Te+^132^I, ^133^I and ^135^I ranged from 0 to 13.3 Gy (AM: 0.34 Gy, median: 0.062 Gy), and internal thyroid doses from inhalation of ^131^I, ^132^Te+^132^I, ^133^I and ^135^I ranged from 0 to 0.28 Gy (AM: 0.046 Gy, median: 2.8 × 10^−3^ Gy). The AM of thyroid doses from all exposure pathways was 0.43 Gy (range from 3.5 × 10^−4^ Gy to 13.7 Gy) and the median was 0.13 Gy. The highest thyroid doses were received by cohort members after test #2 conducted on 24 September 1951 (AM: 1.1 Gy, geometric mean (GM): 0.70 Gy), followed by test #1 conducted on 29 August 1949 (AM: 0.49 Gy, GM: 0.047 Gy) and the thermonuclear test #4 conducted on 12 August 1953 (AM: 0.16 Gy, GM: 0.14 Gy). The predominant pathway of thyroid exposure in the cohort was intake of ^131^I with fresh milk from mares and cows, and dairy products made from these types of milk. Although the uncertainties in the dose estimates were not quantified, it was estimated that they are characterized by a geometric standard deviation from 2.0 to 4.0 for most individuals. The study cohort received quite high thyroid doses compared to other populations exposed to fallout from the Chernobyl accident and atmospheric nuclear weapons tests conducted elsewhere. The cohort included individuals exposed *in utero*, as children and as adolescents to high doses of radiation to the thyroid gland. Consequently, it provides a unique opportunity to assess radiation-related risks of thyroid cancer, thyroid nodules, and other structural and functional non-cancer thyroid diseases.

## Introduction

Ionizing radiation is a well-established human carcinogen, with the thyroid gland being a highly radiosensitive organ. Increased rates of thyroid cancer, thyroid nodules and other thyroid diseases associated with external irradiation and internal exposure from intake of radioiodine and radiotellurium isotopes (^131^I, ^132^Te+^132^I, ^133^I, and ^135^I) have been observed in populations affected by atmospheric nuclear weapon tests and nuclear reactor accidents (e.g., Cahoon et al. 2024; Hatch et al. 2019; Lyon et al. 2006; Ostroumova et al. 2009; Tronko et al. 2017; de Vathaire et al. 2023; Zablotska et al. 2011).

Between 1949 and 1962, more than 100 atmospheric nuclear weapons tests were conducted at the Semipalatinsk Nuclear Test Site (SNTS) in northeastern Kazakhstan. In 1998, the Scientific Research Institute of Radiation Medicine and Ecology of the Semey Medical University (SRIRME, SMU, Semey, Kazakhstan) and the National Cancer Institute (Bethesda, MD, USA) conducted a joint study to evaluate the prevalence of thyroid nodules detected by ultrasound in a cohort of 2,994 individuals who resided around the SNTS in 1949–1962 (Land et al. 2008). Thyroid doses were reconstructed in deterministic mode for the entire cohort (Land et al. 2008) and in stochastic mode with accounting for shared and unshared uncertainties (Land et al. 2015). During the second dose assessment, the cohort size was reduced from 2,994 to 2,376 individuals, removing 618 individuals with uncertain residence histories.

SRIRME has recently resumed the cohort follow-up by repetition of an ultrasound examination of the thyroid gland in the alive cohort members and linkage to a regional population-based cancer registry to identify thyroid cancer cases in the entire cohort. The cohort size increased to 3,183 participants by adding 189 individuals who underwent an ultrasound examination in 1999–2002 in the SRIRME clinic. Moreover, the residence histories of all cohort members were checked and verified. A detailed state-of-the-art methodology for estimating external and internal doses from the fallout after atmospheric nuclear weapons tests including the inhalation pathway has been published (Anspaugh et al. 2022; Beck et al. 2022; Bouville et al. 2022; Melo et al. 2022; Simon et al. 2022; Thiessen et al. 2022). Thus, it became necessary to reassess thyroid doses to the cohort. This paper describes the methodology, input data and results of the reconstruction of individual thyroid doses in a cohort of individuals who had been exposed to fallout after some atmospheric nuclear weapons tests conducted at the SNTS in 1949–1962.

## Materials and methods

### Atmospheric nuclear weapons testing at the SNTS

Among the atmospheric nuclear weapons tests conducted at the SNTS between 1949 and 1962, Gordeev et al. (2002) identified 11 tests that resulted in the exposure of residents of settlements in northeastern Kazakhstan. In addition, the present study considered four tests that resulted in the exposure of cohort members who either lived in the Degelen railway station (next to the city of Kurchatov) and were exposed to fallout after tests #6, #8, and #32, and or those who lived in the city of Semipalatinsk (now Semey) and were exposed to fallout after test #16. Table 1 summarizes the characteristics of the 15 tests that were considered in the present study.

**Table 1:** Characteristics of tests conducted at the semipalatinsk nuclear test site that contributed to the exposure of the local population (based on Doobasov et al. 1994; Gordeev et al. 2002)

Test #	Test date (dd/mm/yyyy)	Height above ground, $\:H$ (m)	Total yield, $\:q$ (kt)	Maximum height of radioactive cloud, $\:{H}_{max}$ (km)	Deposition velocity of particles with size of 50 μm, $\:{w}_{50}$ (km h^−1^)	Average wind speed, $\:\nu\:$ (km h^−1^)	Fraction of fresh pasture grass, $\:{F}_{grass}$
1	29/08/1949	30	22	8.7	0.73	47.0	1.0
2	24/09/1951	30	38	10.0	0.74	26.4	0.6
4^a^	12/08/1953	30	400	17.9	0.76	64.6	1.0
6	03/09/1953	256	5.8	6.5	0.72	34.2	1.0
8	10/09/1953	268	4.9	6.2	0.72	32.4	1.0
13	05/10/1954	0	4.0	5.7	0.72	43.3	0.25
16	23/10/1954	410	62	11.6	0.74	73.8	0
18	30/10/1954	55	10	7.2	0.73	32.9	0
19	29/07/1955	2.5	1.3	4.3	0.72	42.0	1.0
20	02/08/1955	2.5	12	7.4	0.73	36.6	1.0
26	16/03/1956	0.4	14	7.7	0.73	39.0	0
28	24/08/1956	93	27	9.2	0.74	71.2	1.0
32	10/09/1956	270	38	10.2	0.74	36.1	1.0
148	07/08/1962	0	9.9	7.1	0.73	10.0/25.2^b^	1.0
172	25/09/1962	0	7.0	6.5	0.72	32.4	0.6

^a^ Thermonuclear test

^b^ Wind speed was 10 km h^−1^ during the first 12 h after the detonation/later wind was 25.2 km h^−1^ (SRIRME 1998)

### Study population

The study cohort comprised people who were mostly younger than 21 years at first exposure and resided in villages adjacent to the SNTS. Details of cohort construction were published elsewhere (Land et al. 2008). In brief, 2,670 out of 3,183 (83.9% of the total) individuals were born before 29 August 1949, the date of the first atmospheric nuclear weapons test conducted at the SNTS, and their median age at the time of first exposure was 10.8 y. The remaining cohort members were born between 29 August 1949 and 5 December 1962, including 58 individuals who were exposed *in utero*, and individuals exposed after birth with a median age at first exposure of 2.9 y.

The residence histories of all study participants were verified and confirmed using the State Scientific Automated Medical Registry, created in 2003 and maintained by SRIRME scientists (Apsalikov et al. 2019). It was confirmed that the cohort members lived during the testing period in 1949–1962 in 146 settlements located in Abai, East-Kazakhstan, and Pavlodar Oblasts^1^^,^^2^ in the northeast of Kazakhstan, as well as in the neighboring Altai Krai of the Russian Federation. Of the 3,183 individuals, 2,655 (83.4% of the total) did not leave their places of permanent residence in 1949–1962, while 467 individuals changed their place of residence once, 55 did it twice, and six individuals changed their place of residence three or more times.

### Assessment of radiation doses

The present study used the so-called joint U.S.-Russian methodology to calculate thyroid doses from external irradiation, ingestion of radionuclides with locally produced milk and dairy products, and inhalation of contaminated air during the passage of a radioactive cloud (Anspaugh et al. 2022; Beck et al. 2022; Bouville et al. 2022; Gordeev et al. 2006a, b). These exposure pathways are associated with different periods of dose accumulation. For example, 95% of external dose was delivered within the first year after detonation, while 5% due to ^137^Cs was delivered during the following decades. The internal thyroid dose from inhalation of radioiodine isotopes was realized within a few weeks after passage of a radioactive cloud, whereas thyroid exposure due to ingestion lasted up to three months.

The U.S.-Russian methodology used the exposure rate at 12 h post-detonation, H + 12, $\:\dot{X}\left(12\right)$, and the time of arrival (TOA) of fallout as the basis for dose calculations. The parameter values of the dose reconstruction model have been adjusted to local exposure conditions where possible. Details of the methodology, input data, and behaviour and dietary data for the cohort members used for dose calculations are given in the sections below.

### Exposure rate at H + 12 and time of arrival of fallout

Whenever possible, $\:\dot{X}\left(12\right)$ values were derived from the exposure rate measured in or around the settlement that were found in historical reports and in literature (e.g., Archive 1994; Gordeev 1999, 2001a, b; Gordeev et al. 2002, 2006a, b; Loborev et al. 1994, 1997; Logachev 1997; Shoikhet et al. 1997, 2002; SRIRME 1998). The $\:\dot{X}\left(12\right)$ value was estimated from the exposure rate measured at time $\:t$ after detonation using a ten-term exponential function (Beck et al. 2022; Henderson 1991):

1 $$\:\dot{X}\left(12\right)=\dot{X}\left(t\right)/\sum_{i=1}^{i=10}{a}_{i}\cdot{e}^{{L}_{i}\cdot\:t}$$

where $\:\dot{X}\left(t\right)$ is the exposure rate at time $\:t$ after detonation (mR h^−1^); $\:{a}_{i}$ (unitless) and $\:{L}_{i}$ (h^−1^) are parameters of the fitting function.

Following each detonation, exposure rates were measured in a given settlement if it had been potentially exposed to fallout: such measurements were carried out in only 35 of the 146 settlements where cohort members resided in 1949–1962. In the 111 settlements not covered by measurements, exposure rates were assessed using developed approaches to reconstruct exposure rates along and across the fallout trace based on exposure rates measured after the same test at 66 neighboring settlements, including 31 settlements where cohort members did not reside during the testing period. The available data and the interpolations in space and time that were used to reconstruct the exposure rates at H + 12 in all settlements considered in the study are described in detail in (Drozdovitch et al. 2025). As a result, the exposure rates at H + 12 were reconstructed for 97 settlements, while 14 settlements were considered unexposed because they were located far away from the fallout trace.

TOA values (in hours) were obtained from historical reports and literature. If a TOA value was not reported for a given location and test, it was estimated using the following equation:

2 $$\:{t}_{TOA}=x/\nu\:$$

where $\:{t}_{TOA}$ is the time of arrival of fallout (h); $\:x$ is the distance between the detonation site along the axis of the fallout trace and the settlement (km); and $\:\nu\:$ is the average wind speed given in Table 1 (km h^−1^).

The average wind speed was estimated based on the TOA observed on the fallout trace at known distances from a detonation site. It was assumed that for a given test the wind had the same average speed in the layer from the radioactive cloud top to the surface of the earth and during the time when the cloud was spreading. The average wind speed values according to (Gordeev et al. 2002) are given in Table 1.

**Fig. 1:**
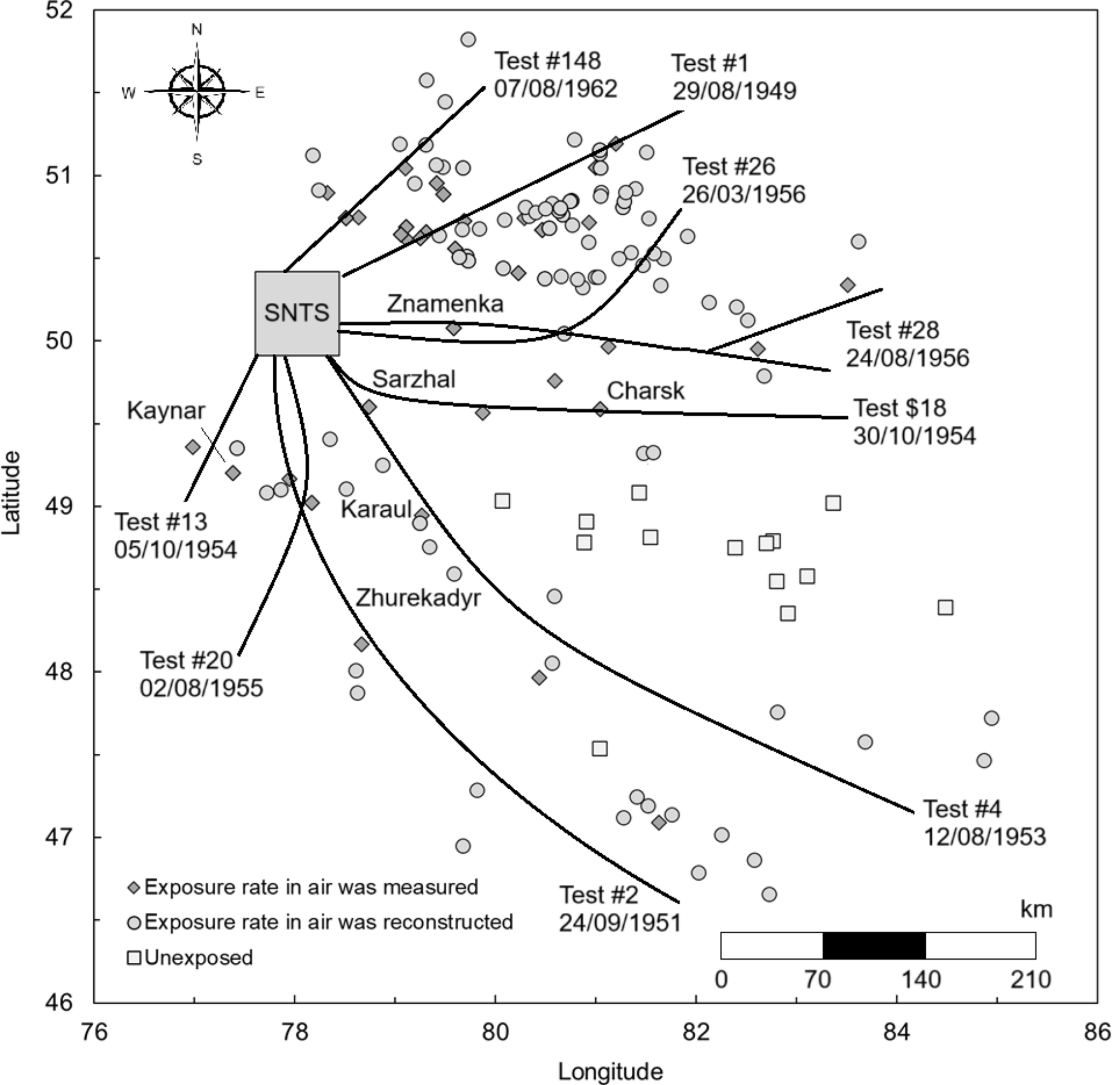
Location of settlements where the investigated cohort members resided during atmospheric nuclear weapons testing in 1949–1962. The fallout trajectories for nine selected tests (out of the 15 tests considered in this study – see Table 1) conducted at the Semipalatinsk Nuclear Test Site (SNTS) are shown schematically based on (Gordeev et al. 2001b 2002)

Figure 1 schematically shows the location of the settlements where the cohort members resided during atmospheric nuclear weapons testing in 1949–1962, indicating the settlements where the exposure rate was measured (*n* = 35) or reconstructed (*n* = 97), as well as unexposed settlements (*n* = 14). The figure also shows the trajectories of selected radioactive fallout (based on Gordeev et al. 2001b 2002).

### Thyroid doses from external irradiation

To estimate thyroid doses from external irradiation from fallout after atmospheric nuclear weapons test Eq. 3 was used (Bouville et al. 2022):

3 $$\:{D}_{k}^{ext}=\dot{X}\left(12\right)\cdot IF\cdot {C}_{k}\cdot {BF}_{k}$$

where $\:{D}_{k}^{ext}$ is the thyroid dose from external irradiation to a cohort member who was in age group $\:k$ at the time of the test (Gy); $\:IF$ is the integral from TOA to the time of the end of residence in the settlement of the exposure rate normalized to $\:\dot{X}\left(12\right)$ = 1 mR h^−1^ (h); $\:{C}_{k}$ is the age-dependent conversion coefficient from exposure in air to thyroid dose given in Table 2 according to (Bouville et al. 2022) (Gy mR^−1^). Because 95% of the external dose was delivered within the first year after detonation, any change in the conversion coefficient due to cohort aging was not taken into account; $\:{BF}_{k}$ is the behavioural factor that takes into account occupancy factors, describing the fraction of time spent by an individual of age group $\:k$ in different types of places in the village (house, school, outdoors), and location factors, which are defined as the ratio of the dose rate in different types of places in the village to the dose rate on undisturbed ground away from any buildings (unitless). Detailed information on how the values of the behavioural factor, $\:{BF}_{k}$, were calculated is presented in the subsection titled “Behavioural factor for external irradiation”.

**Table 2:** Age-dependent parameters of the thyroid dose reconstruction model used in the present study for external irradiation

Age (y)	Conversion coefficient, $\:{C}_{k}$ (Gy mR^−1^)	Time spent indoors (h)			
		school in session		school not in session	
		Kazakh	Russian	Kazakh	Russian
*In utero*	6.6 × 10^−6^	20.0^a^	20.0^a^	16.0^a^	16.0^a^
0–0.99	8.6 × 10^−6^	22.4	23.5	23.3	23.6
1–2	8.6 × 10^−6^	19.0	15.5	19.0	15.0
3–7	7.9 × 10^−6^	18.8	15.7	15.8	14.7
8–12	7.9 × 10^−6^	15.0/4.5^b^	14.0/4.5^b^	14.0	13.5
13–17	7.3 × 10^−6^	13.5/5.7^b^	14.2/5.7^b^	12.2	13.2
Adults	6.6 × 10^−6^	16.0/20.0^c^	16.0/20.0^c^	10.0/18.0^c^	10.0/18.0^c^

^a^ Pregnant woman

^b^ In residential houses/at schools

^c^ Agricultural workers/office workers

The integral of the exposure rate normalized to $\:\dot{X}\left(12\right)$ = 1 mR h^−1^ from TOA to the time of the end of residence in the settlement, *TC*, was calculated as shown in Eq. 4:

4 $$\:IF=\sum_{i=1}^{i=10}\frac{{a}_{i}}{{L}_{i}}\cdot \left({{e}^{{L}_{i}\cdot\:{t}_{TOA}}-e}^{{L}_{i}\cdot\:TC}\right)$$

    

By analogy with (Land et al. 2008; Simon et al. 2006a), the following tests were used to characterize the tests conducted at the SNTS:

- First atmospheric nuclear weapons test Trinity conducted in New Mexico on 16 July 1945 (Bouville et al. 2020) was used as a surrogate for test #1 conducted at the SNTS.
- Test Tesla conducted at Nevada Test Site on 1 March 1955 (Beck et al. 2022) was used for all other tests conducted at the SNTS considered in the present study.

The fitting parameters, $\:{a}_{i}$ and $\:{L}_{i}$, of a ten-term exponential function describing the change of the exposure rate for tests Trinity and Tesla were taken from (Henderson 1991).

### Thyroid doses from ingestion of ^131^I and short-lived radioiodine and radiotellurium isotopes

The present study considered the consumption of the following types of milk and dairy products: fresh cow milk, cow milk in tea, and sour cow milk. In addition, for individuals of Kazakh ethnicity, the consumption of mare milk and koumiss, which is fermented mare milk, was also considered. The study did not consider consumption of goat milk and sheep milk, as only very few cohort members reported their consumption.

The thyroid dose from ingestion of ^131^I, ^132^Te+^132^I, ^133^I, and ^135^I with different types of milk and dairy products^3^ was calculated as in Eq. 5:

5 $$\:{D}_{n,j,k}^{ing}=\sum_{i}{A}_{j,i}^{milk,TIA}\cdot{PF}_{j,i}\cdot{V}_{n,i}\cdot{DC}_{j,k}^{ing}$$

where $\:{D}_{n,j,k}^{ing}$ is the thyroid dose from ingestion of radionuclide *j* for a cohort member $\:n$ of age group $\:k$ at the time of a test (Gy); $\:{A}_{j,i}^{milk,TIA}$ is the time-integrated activity of radionuclide *j* in foodstuff *i* (cow or mare milk and dairy products) (Bq L^−1^ d); $\:{PF}_{j,i}$ is the processing factor reflecting the time change in the activity of radionuclide *j* in foodstuff *i* compared to fresh milk due to radioactive decay between production and consumption of the food product (unitless); $\:{V}_{n,i}$ is the daily consumption of foodstuff *i* by a cohort member $\:n$ (L d^−1^); and $\:{DC}_{j,k}^{ing}$ is the ingestion thyroid dose coefficient for radionuclide *j* corresponding to age group *k* given by Melo et al. (2022) (Gy Bq^−1^).

#### Activity concentration in milk

The time-integrated activity concentration of radioiodine and radiotellurium isotopes in milk was calculated as in Eq. 6 (Müller and Pröhl 1993):

6 $$\begin{aligned}{A}_{j,i}^{milk,TIA}=\:&{A}_{j}^{grass}\left({t}_{TOA}\right)\cdot\:{I}_{a}\cdot{F}_{grass}\cdot\:{TF}_{a,j}\\&\cdot\:\frac{{\lambda\:}_{b}^{j}}{({\lambda\:}_{w}^{j}+{\lambda\:}_{r}^{j})\cdot\:({\lambda\:}_{b}^{j}+{\lambda\:}_{r}^{j})}\end{aligned}$$

where $\:{A}_{j}^{grass}\left({t}_{TOA}\right)$ is the activity concentration of radionuclide *j* in pasture grass at TOA (Bq kg^−1^); $\:{I}_{a}$ is the intake rate of pasture grass by type *a* of dairy animal, cow or mare (kg d^−1^); $\:{F}_{grass}$ is the fraction of fresh pasture grass available for feeding cows and mares (unitless); $\:{TF}_{a,j}$ is the feed-to-milk transfer coefficient of radionuclide *j* for type *a* of dairy animals (d L^−1^); $\:{\lambda\:}_{b}^{j}$ is the biological elimination rate of a stable element (either iodine or tellurium) from milk (d^−1^); $\:{\lambda\:}_{w}^{j}$ is the elimination rate of a stable element (either iodine or tellurium) from grass due to weathering and growth dilution (d^−1^); $\:{\lambda\:}_{r}^{j}$ is the radioactive decay constant of radionuclide *j* (d^−1^).

Note that the intake rate of fresh contaminated pasture grass was obtained as $\:{I}_{a}\cdot\:{F}_{grass}$. According to Drozdovitch et al. (2011), the intake rate of pasture grass by a cow, $\:{I}_{cow}$, was 16 kg d^−1^ and 15 kg d^−1^ during July–September and October, respectively, in villages where the predominant ethnic group was Kazakh, and 20 kg d^−1^ and 15 kg d^−1^ for the same time periods in villages where the predominant ethnic group was Russian. The intake rate of pasture grass by mare, $\:{I}_{mare}$, was 19 kg d^−1^ and 25 kg d^−1^ in villages where the predominant ethnic group was Kazakh and Russian, respectively. The difference in grass consumption primarily reflects the lower availability of grass in pastures located south of the Irtysh River, where the predominant ethnic group in the villages was Kazakh, compared to pastures located north of the Irtysh River, where the predominant ethnic group in the villages was Russian. The fraction of fresh pasture grass available for feeding cows and mares, $\:{F}_{grass}$, depended on the season. In July–August, the animals were on pasture, and pasture grass was fully available, i.e., $\:{F}_{grass}$=1.0. From September to mid-October, the fraction of fresh pasture grass available for feeding decreased due to the reduced growth of pasture vegetation, and dairy animals were given supplementary feed. By mid-October there was practically no pasture grass and snow cover began. Consequently, the cows were moved to stall keeping, and the mares and horses were moved to distant pastures 30–100 km from the village, i.e., $\:{F}_{grass}$=0 for the period from mid-October to April. Table 1 shows the $\:{F}_{grass}$-values for each considered test.

Table 3 provides the radionuclide-specific values of the processing factor, the weathering and biological elimination rates, the radioactive decay constant as well as the feed-to-milk transfer coefficient. The feed-to-milk transfer coefficient of short-lived ^133^I and ^135^I was calculated from the feed-to-milk transfer coefficient of ^131^I as in Eq. 7 (NCRP 1996):

**Table 3:** Radionuclide-specific parameters of the thyroid dose reconstruction model for internal irradiation

Parameter	Units	Symbol	Eq.	^131^I	^132^Te+^132^I^a^	^133^I	^135^I	Reference
Processing factor: cow, mare milk	unitless	$\:{PF}_{j,milk}$	(5)	0.96	0.90	0.67	0.28	This study
Processing factor: sour milk, koumiss	unitless	$\:{PF}_{j,dairy}$	(5)	0.92	0.81	0.45	0.08	This study
Radioactive decay constant	d^−1^	$\:{\lambda\:}_{r}^{j}$	(6, 27)	0.0864	0.216	0.80	2.52	(Eckerman and Endo 2008)
Weathering rate	d^−1^	$\:{\lambda\:}_{w}^{j}$	(6, 27)	0.069	0.047	0.069	0.069	(Thiessen et al. 2022)
Biological elimination rate	d^−1^	$\:{\lambda\:}_{b}^{j}$	(6, 27)	0.99	0.69	0.99	0.99	(Müller and Pröhl 1993)
Feed-to-milk transfer coefficient, cow	d L^−1^	$\:{TF}_{j,cow}$	(6, 27)	4.0 × 10^−3^	5.0 × 10^−4^	2.2 × 10^−3^	1.1 × 10^−3^	(Fesenko et al. 2007)
Feed-to-milk transfer coefficient, mare	d L^−1^	$\:{TF}_{j,mare}$	(6, 27)	3.0 × 10^−2^	4.4 × 10^−3b^	1.7 × 10^−2^	8.5 × 10^−3^	—^c^
Activity fraction to total beta-activity at H + 12^d^	unitless	$\:{\left(\frac{{GD}_{j}\left(12\right)}{\beta\:\left(12\right)}\right)}_{R/V=0.5}$	(8)	7.60 × 10^−3^ 9.48 × 10^−3^ 6.72 × 10^−3^	2.16 × 10^−2^ 2.53 × 10^−2^ 1.80 × 10^−2^	8.73 × 10^−2^ 1.04 × 10^−1^ 7.38 × 10^−2^	9.45 × 10^−2^ 1.11 × 10^−1^ 7.87 × 10^−2^	(Bouville et al. 2020) (Beck et al. 2022) (Beck et al. 2022)

^a^ Parameter values are given for parent ^132^Te

^b^ Based on the ratio of transfer coefficient for mare milk to that for cow milk of 8.8 for strontium (Simon S.L., personal communication, 2024)

^c^ Simon S.L., personal communication, 2024

^d^ Upper figure is for test #1; middle figure is for other tests considered in the study, except for the high-altitude tests #6, #8, #16, #32; bottom figure is for high-altitude tests #6, #8, #16, #32

7 $$\:{TF}_{a,j}={TF}_{a,I-131}\cdot\frac{{\lambda\:}_{b}^{j}}{{\lambda\:}_{b}^{j}+{\lambda\:}_{r}^{j}}\:$$

#### Activity concentration in pasture grass

The key assumptions in the U.S.-Russian methodology are that (i) only particles with an activity median aerodynamic diameter (AMAD) of less than 50 μm are biologically active, i.e., they are intercepted, initially retained on vegetation, and finally lead to contamination of dairy products; and (ii) the biologically active fraction those particles in the fallout depends on the test yield, the maximum height of the radioactive cloud, the average wind speed, the distance from the detonation site, and the distance of pastures from the axis of the fallout trace (Anspaugh et al. 2022; Gordeev 1999; Gordeev et al. 2006b). According to this methodology, the concentration of radionuclide *j* in pasture grass at TOA was calculated as given in Eq. 8 (Anspaugh et al. 2022):

8 $$\begin{aligned}\:{A}_{j}^{grass}\left(TOA\right)=&\dot{X}\left(12\right){\cdot\:{\left(\frac{\beta\:\left(12\right)}{\dot{X}\left(12\right)}\right)}_{R/V}\cdot\:\:N}_{50}\\&\cdot\:{\left(\frac{{GD}_{j}\left(12\right)}{\beta\:\left(12\right)}\right)}_{R/V=0.5}{\cdot\:e}^{{\lambda\:}_{r}^{j}\cdot\:\frac{12-{t}_{TOA}}{24}}\cdot\:\alpha\:\end{aligned}$$

where $\:{\left(\frac{\beta\:\left(12\right)}{\dot{X}\left(12\right)}\right)}_{R/V}$ is the ratio of deposited total beta-activity to $\:\dot{X}\left(12\right)$ (Bq m^−2^ per mR h^−1^). This ratio depends on the ratio of the fraction of refractory nuclide activity ($\:R$) and the fraction of volatile nuclide activity ($\:V$) in the total activity deposited on the ground at the considered location, $\:R/V$; $\:{N}_{50}$ is the fraction of total beta-activity deposited on less than 50 μm particles (unitless); $\:{\left(\frac{{GD}_{j}\left(12\right)}{\beta\:\left(12\right)}\right)}_{R/V=0.5}$ is the activity fraction of ground deposition density of radionuclide *j* to the total deposited beta-activity at H+12 for $\:R/V$=0.5 (unitless); $\:{t}_{TOA}$ is the time of arrival of fallout (h). Of note, the same $\:{t}_{TOA}$ was assumed for the village and for the pastures located within 2–5 km around this village; $\:\alpha\:$ is the interception factor, i.e. the fraction of activity on particles with an AMAD of less than 50 μm that was intercepted and retained by pasture grass (unitless).

Equation 9 was used to calculate the fraction of total beta-activity deposited on particles with an AMAD of less than 50 μm, $\:{N}_{50}$, (Beck et al. 2022):

9 $$\:{N}_{50}={N}_{50}^{axis}-1.3\cdot\:\sqrt{{N}_{50}^{axis}}\cdot\:ln\left(\frac{\dot{X}}{{\dot{X}}_{axis}}\right)$$

where $\:{N}_{50}^{axis}$ is the fraction of total beta-activity deposited on particles with an AMAD of less than 50 μm along the axis of the fallout trace (unitless); $\:\dot{X}$ is the exposure rate at the pasture (mR h^−1^); $\:{\dot{X}}_{axis}$ is the exposure rate on the axis of the fallout pattern at the same time as the exposure rate at the pasture (mR h^−1^).

The value of $\:{N}_{50}^{axis}$ was calculated using Eq. 10 (Beck et al. 2022; Gordeev 1999):

10 $$\:{N}_{50}^{axis}=1-\left(1-0.1\cdot\:{e}^{-\frac{44\cdot\:{q}^{0.4}-H}{70}}\right)\cdot\:{e}^{-{\left(1.6\cdot\:\frac{{t}_{TOA}}{{H}_{max}}\cdot\:{w}_{50}\right)}^{3}}$$

where $\:q$ is the total yield of the considered test (kt); $\:H$ is the height of the explosion above the ground (m); $\:{H}_{max}=4.0\cdot\:{q}^{0.25}+H/1000$ is the maximum height of the radioactive cloud (MHRF 2001) (km); and $\:{w}_{50}$ is the deposition velocity of particles with AMAD=50 μm (km h^−1^).

The deposition velocity of particles depended on the maximal height of the radioactive cloud. The $\:{w}_{50}$ values for particles with an AMAD of 50 μm, given for each test in Table 1, were calculated using Eq. 11 fitted to the tabulated data of Gordeev et al. (1994) for silicate soil with a density of 2.5 g cm^−3^, which was typical for the SNTS:

11 $$\begin{aligned}\:{w}_{50}=&-5.73\cdot\:{10}^{-5}\cdot\:{{H}_{max}}^{2}\\&+4.67\cdot\:{10}^{-3}\cdot\:{H}_{max}+0.697\end{aligned}$$

Equation 12 was used to calculate the interception factor α_dry_ for steppe pasture grass in Kazakhstan for dry deposited radionuclides (Gordeev 1999):

12 $$\:{\alpha\:}_{dry}=0.6\cdot\:b$$

where $\:b$=0.42 is the steppe grass mass (kg m^−2^).

As was shown in (Drozdovitch et al. 2025), the eastern part of the fallout trace after test #28 could have been formed by wet deposition. In the case of wet deposition, the interception factor was calculated as given in Eq. 13:

13 $$\:{\alpha\:}_{wet}={CF}_{wet}\cdot\:{\alpha\:}_{dry}$$

where $\:{CF}_{wet}$=0.2 is the correction factor which accounts for the fact that the fraction of radioactivity intercepted by vegetation was smaller during fallout with rain than that for dry fallout because of wash-out of particles from vegetation leaves (Hoffman et al. 1992). The $\:{CF}_{wet}$ value was derived from the data on dry and wet interception factor values summarized by Pröhl (2009).

#### Test-related parameter values

Table 3 provides the ratio of the activity fraction of ground deposition density of radionuclide *j* to the total beta-activity at H + 12, $\:{\left(\frac{{GD}_{j}\left(12\right)}{\beta\:\left(12\right)}\right)}_{R/V=0.5}$. Table 4 provides the ratio of deposited total beta-activity to exposure rate at H+12, $\:{\left(\frac{\beta\:\left(12\right)}{\dot{X}\left(12\right)}\right)}_{R/V}$, that depends on the $\:{N}_{50}$-values and corresponding $\:R/V$-values. The values of both parameters are given separately for test #1 and all other tests considered in the study as described above.

**Table 4:** Ratio of deposited total beta-activity to exposure rate at H + 12, $\:{\left(\frac{\beta\:\left(12\right)}{\dot{X}\left(12\right)}\right)}_{R/V}$, corresponding to the $\:{N}_{50}$ and $\:R/V$ values. AMAD - activity median aerodynamic diameter

Fraction of total beta-activity deposited on particles with an AMAD of less than 50 μm, $\:{N}_{50}$	$\:R/V$	$\:{\left(\frac{\beta\:\left(12\right)}{\dot{X}\left(12\right)}\right)}_{R/V}$(Bq m^−2^ per mR h^−1^) for test	
		#1^a^	Other tests^b^
> 0.83	0.5	4.11 × 10^6^	3.64 × 10^6^
0.43–0.83	1.0	4.92 × 10^6^	4.21 × 10^6^
0.23–0.43	1.5	5.43 × 10^6^	4.59 × 10^6^
0.09–0.23	2.0	5.79 × 10^6^	4.87 × 10^6^
< 0.09	3.0	6.24 × 10^6^	5.25 × 10^6^

^a^ For test Trinity conducted in New Mexico on 16 July 1945 (Bouville et al. 2020)

^b^ For test Tesla conducted at Nevada Test Site on 1 March 1955 (Beck et al. 2022)

The U.S.-Russian methodology was developed for low-altitude nuclear detonations. However, tests #6, #8, #16 and #32 were conducted at an altitude of 256–410 m and, thus, these tests were high-altitude tests. Beck et al. (2022) suggested that the methodology for low-altitude detonations can also be applied to high-altitude detonations with the following modifications: (i) there was no significant fractionation ($\:R/V$ =1.0) and (ii) the AMAD of all particles in the fallout was less than 20 μm. These modifications were used to calculate internal thyroid doses for individuals resided in settlements affected by tests #6, #8, #16 and #32.

### Thyroid doses from inhalation of ^131^I and short-lived radioiodine and radiotellurium isotopes

The thyroid dose arising from inhalation of radionuclide *j* with contaminated air was calculated according to (Anspaugh et al. 2022) using Eq. 14:

14 $$\begin{aligned}\:{D}_{j,k}^{inh}=&\sum_{l}\dot{X}\left(12\right)\cdot\:{\left(\frac{\beta\:\left(12\right)}{\dot{X}\left(12\right)}\right)}_{R/V}\cdot\:{N}_{l}\left({N}_{50}\right)\\&\cdot\:{\left(\frac{{GD}_{j}\left(12\right)}{\beta\:\left(12\right)}\right)}_{{R/V}^{{\prime\:}}}{\cdot\:e}^{{\lambda\:}_{r}^{j}\cdot\:\frac{12-{t}_{TOA}}{24}}\cdot\:{RF}_{k}\cdot\:{BR}_{k}\\&\cdot\:\frac{1}{{v}_{d}\left({N}_{50}\right)}\cdot\:{DC}_{j,k}^{inh}\end{aligned}$$

where $\:{D}_{j,k}^{inh}$ is the thyroid dose from inhalation of radionuclide *j* for a cohort member of age group $\:k$ at the time of a nuclear test (Gy); $\:{N}_{l}\left({N}_{50}\right)$ is the fraction of total deposited beta activity on 1–20 μm ($\:l$=1), 20–100 μm ($\:l$=2), and > 100 μm ($\:l$=3) particles that depends on $\:{N}_{50}$ (unitless); $\:{\left(\frac{{GD}_{j}\left(12\right)}{\beta\:\left(12\right)}\right)}_{{R/V}^{{\prime\:}}}$ is the activity fraction of ground deposition density of radionuclide *j* to the total deposited beta-activity at H + 12 depending on $\:R/V$ (unitless); $\:{RF}_{k}$ is the reduction factor of inhaled radioactivity due to indoor occupancy (unitless); $\:{BR}_{k}$ is the breathing rate of a representative individual of age group $\:k$ (m^3^ h^−1^) given by (ICRP 2002); $\:{v}_{d}\left({N}_{50}\right)$ is the deposition velocity for a given particle size that depends on $\:{N}_{50}$ (m h^−1^); $\:{DC}_{j,k}^{inh}$ is the thyroid dose coefficient for radionuclide *j* for an individual of age group *k* (Gy Bq^−1^) for inhalation of particles with an AMAD of less than 20 μm or for ingestion of particles with an AMAD of more than 20 μm (Melo et al. 2022).

The values of $\:{N}_{l}\left({N}_{50}\right)$ and $\:\frac{1}{{v}_{d}\left({N}_{50}\right)}$ were calculated using the regression equations given by Figs. B3 and B4 in (Anspaugh et al. 2022), respectively. The dose calculation for particles with an AMAD of 20–100 μm involved a reduction coefficient of 0.4 that represents an average fraction of particles inhaled through the nose and deposited in the nasopharynx or particles inhaled through the mouth and deposited there or in the oropharynx that are generally swallowed, while all the activities on particles with an AMAD larger than 100 μm are ingested (Anspaugh et al. 2022). As mentioned above, for high altitude tests #6, #8, #16 and #32, it was assumed that all particles in the fallout were less than 20 μm in AMAD.

### Behaviour and dietary data for the cohort members

#### Behavioural factor for external irradiation

The age-dependent behavioural factor, *BF*_*k*_ (Eq. (3)), was calculated as:

15 $$\begin{aligned}{BF}_{k}=&[{LF}_{out}\cdot\:(24-{T}_{k}^{in,\:house}-{T}_{k}^{in,\:school})\\&+{LF}_{in}^{house}\cdot\:{T}_{k}^{in,house}+{LF}_{in}^{school}\cdot\:{T}_{k}^{in,\:school}]/24\end{aligned}$$

where $\:L{F}_{out}$ is the outdoor location factor assumed to be equal to 1.0, for lack of information on where in the village the exposure-rate measurements were carried out (unitless), $\:{LF}_{in}^{house}$ and $\:{LF}_{in}^{school}$ are the location factors for indoors occupancy for staying in house and school (office building for adults), respectively (unitless); $\:{T}_{k}^{in,\:house}$ and $\:{T}_{k}^{in,\:school}$ is the daily time spent indoors at house or in school (office building) by a person of age group $\:k$, respectively (h).

The daily time spent indoors per day was obtained using an interview data collection methodology by a focus group for representative individuals of the following ages who resided around the SNTS: < 1 year old, 1–3 y, 4–6 y, 7–14 y, and 15–21 y (Drozdovitch et al. 2011; Schwerin et al. 2010). Data on daily time spent indoors were collected for two seasons of the year: the months of June–August when school was not in session, and the rest of the year when school was in session. The present study used the focus groups data on daily time spent indoors by individuals of Kazakh and Russian ethnicity that were adjusted to the ICRP age groups: < 1 year old, 1–2 y, 3–7 y, 8–12 y, 13–17 y, and adults. For adults, the daily time spent indoors depends on the person’s profession. For agricultural workers who worked mainly outdoors, such as agronomists, machine operators, or tractor drivers, the daily time spent indoors was taken to be 10 h in June–August and 16 h the rest of the year, while for office workers, such as teachers, accountants, or medical doctors, this time was taken to be 18 h and 20 h, respectively. Table 2 provides the age-dependent values of daily time spent indoors used in the present study.

The location factor for indoors occupancy was determined by the construction material of the corresponding house or school (office building) and was calculated as $\:L{F}_{in}=1/SF$, where $\:SF$ is the shielding factor for adobe ($\:{SF}_{adobe}$=13), brick ($\:{SF}_{brick}$=10, for school and office buildings only), and wooden buildings ($\:{SF}_{wood}$=3), respectively (Gordeev et al. 2002; MHRF 2001). Because information on the construction material of individual houses of study participants was not available, the settlement-specific $\:{LF}_{in}^{house}$-value for individuals of Kazakh or Russian ethnicity was calculated for each settlement of residence as follows:

16 $$\:{LF}_{in}^{hous}={F}_{wood}/{SF}_{wood}+(1-{F}_{wood})/{SF}_{adobe}$$

where $\:{F}_{wood}$ is the fraction of individuals of Kazakh or Russian ethnicity who lived in wooden houses in the settlement (unitless).

Information on the construction material of schools and the fraction of individuals of Kazakh and Russian ethnicity living in wooden houses during the testing period in 1949–1962 was collected for all 146 settlements, where cohort members resided, through contacts with the local authorities and interviews of senior residents during the present study as well as during a focus groups study. Table 5 shows the fraction of individuals of Kazakh and Russian ethnicity who lived in wooden houses, $\:{F}_{wood}$, the construction material of schools, and the values of behaviour factors calculated using Eqs. (15) and (16), as example, for a 10-y-old resident of selected settlements in Abai, Beskaragai, Borodulikha and Zhanasemey raions of Abai Oblast. During the testing period, most of study cohort participants (2,793 out of 3,183, 87.7% of the total) lived in these four raions.

**Table 5:** Construction material of schools, fraction of individuals of Kazakh and Russian ethnicity who lived in wooden houses, and behaviour factor values calculated using Eqs. (15) and (16) for a 10-y old individual

Raion	Settlement	Fraction of individuals who lived in wooden houses, $\:{F}_{wood}$		School building material^a^	Behaviour factor, $\:BF$, for a 10-y old individual	
		Kazakh	Russian		Kazakh	Russian
Abai	Karaul	0	0	Adobe	0.25/0.46^b^	0.29/0.48
Abai	Sarzhal	0	0	Adobe	0.25/0.46	0.29/0.48
Beskaragai	Bodene	0	0.20	Wood^c^	0.30/0.46	0.37/0.51
Beskaragai	Dolon	0.25	0.95	Wood	0.34/0.50	0.48/0.62
Beskaragai	Ernazar	0.95	0.95	Wood	0.45/0.60	0.48/0.62
Borodulikha	Andronovka	0.25	0.25	Adobe	0.29/0.50	0.33/0.52
Borodulikha	Borodulikha	0.50	0.80	Wood	0.38/0.54	0.46/0.60
Borodulikha	Novopokrovka	0.25	0.25	Brick	0.29/0.50	0.33/0.52
Zhanasemey	Znamenka	0	0	Adobe	0.25/0.46	0.29/0.48
Zhanasemey	Klimentievka	0.20	0.80	Wood	0.33/0.49	0.46/0.60
Zhanasemey	Chagan	0.20	0.20	Adobe^d^	0.28/0.49	0.32/0.51

^a^ All schools were single-story

^b^ School in session/school not in session

^c^ Primary school

^d^ In 1949–1960. Brick school has been constructed in 1961

#### Consumption rates of milk, dairy products and leafy vegetables for ingestion pathway

Table 6 presents data on age-dependent daily consumption of foodstuffs in 1949–1962, obtained during a focus groups study (Drozdovitch et al. 2011). Note that the data on ‘consumptions from the focus groups study’ in the table represent the consumption rates among consumers, not the average consumption rate in the entire age group.

**Table 6:** Consumption rates of milk and dairy products used in thyroid dose calculations (based on (Drozdovitch et al. 2011)

Age/population groups	Consumption rate (L d^−1^) by Kazakhs					Consumption rate (L d^−1^) by Russians		
	Cow milk	Cow milk with tea	Mare milk	Koumiss	Sour milk	Cow milk	Cow milk with tea	Sour milk
*Consumptions from the focus groups study*								
0–0.99 y	0.18		–^a^	–^a^	0.03	0.40		0.045
1–2 y	0.23		0.30	0.10	0.18	0.43		0.065
3–7 y	0.35		0.34	0.22	0.21	0.54		0.08
8–12 y	0.37		0.35	0.24	0.35	0.45		0.10
13–17 y	0.35		0.62	0.36	0.30	0.60		0.09
Adults	0.33		0.80	0.44	0.26	0.70		0.08
*Consumptions calculated using* Eqs. (18), (21),(24)								
0–0.99 y		–^a^					–^a^	
1–2 y		0.005					0.035	
3–7 y		0.03					0.04	
8–12 y		0.04					0.045	
13–17 y		0.06					0.06	
Adults		0.07					0.07	
Pregnant women	0.39	0.19	0.28	0.44	–^b^	0.92	0.09	–^b^
Lactating women	0.075	0.19	0.027	0.20	–^b^	0.23	0.09	–^b^

^a^ Did not consume

^b^ Data were not collected

To assign individual consumption rates to cohort members, questionnaire data collected by means of personal interviews during the ultrasound examination in 1998 (Land et al. 2008) were used. During the personal interviews, information was collected from each cohort member on his/her consumption (yes/no) and frequency of consumption (daily, weekly, rarely) of fresh cow milk, sour cow milk, mare milk and koumiss during the testing period. The frequency of consumption, which was reported by the cohort member, was combined with the consumption rates obtained from the focus groups study to assign individual daily consumptions in the following way:

17 $$\:{V}_{n,i}={V}_{i}\cdot\:{f}_{n,i}$$

where $\:{V}_{n,i}$ is the individual consumption rate of foodstuff *i* by cohort member $\:n$ (L d^−1^); $\:{V}_{i}$ is the average consumption rate of foodstuff *i* obtained from the focus groups study (L d^−1^) (Table 6); and $\:{f}_{n,i}$ is the frequency of consumption of foodstuff *i* reported by a cohort member $\:n$: $\:{f}_{n,i}$=1.0, 0.143, 0.067 and 0.0 for consumption every day, once in a week, rarely (once in two weeks), and no consumption, respectively.

During the focus groups study in 2007, it was recognized that local food habits of drinking cow milk with tea may also be an important source of thyroid exposure from ingestion. Because personal interviews in 1998 did not collect information on the frequency of the consumption of cow milk with tea, daily consumption of cow milk with tea was imputed with age-specific consumption calculated as:

18 $$\:{V}_{k,tea}={P}_{cons,\:k}\cdot\:{V}_{k,\:tea}^{*}$$

where $\:{V}_{k,tea}$ is the consumption rate of cow milk with tea by a cohort member of age group $\:k$ used in the present study (L d^−1^) (Table 6); $\:{P}_{cons,k}$ is the fraction of consumers of cow milk with tea among individuals of age group $\:k$, as reported by focus groups (Drozdovitch et al. 2011) (unitless); and $\:{V}_{k,tea}^{*}$ is the consumption rate of cow milk with tea by individuals of age group $\:k$ averaged among individuals with non-zero consumption reported during focus groups study (L d^−1^).

According to the focus groups study, leafy vegetables were not consumed in the 1950 s in villages predominantly inhabited by Kazakhs because there was no tradition of growing and eating such vegetables. In contrast, in predominantly Russian villages, leafy vegetables, mainly wild sorrel, were eaten during the growing season, usually from May to early July (Drozdovitch et al. 2011). Outside the season, however, leafy vegetables were not available due to the dry weather conditions typical in northeastern Kazakhstan. Since all tests considered in the present study were conducted after the end of July, leafy vegetables consumption was not considered.

#### Reduction factor for inhalation pathway

The age-dependent reduction factor, $\:{RF}_{k}$, was calculated by analogy with the behaviour factor for two seasons of the year as follows:

19 $$\:{RF}_{k}=({F}_{out}\cdot\:(24-{T}_{k}^{in})+{F}_{in}\cdot\:{T}_{k}^{in})/24$$

where $\:{F}_{out}$ = 1.0 is the factor for outdoors occupancy (unitless), $\:{F}_{in}$ is the reduction factor for indoors occupancy (unitless), calculated as $\:{F}_{in}=1/{f}_{in}$, where $\:{f}_{in}$=3 is the reduction factor of radionuclide activity in air, relative to outdoor air, due to indoor occupancy; and $\:{T}_{k}^{in}$ is the daily time spent indoors by a cohort member of age group $\:k$ (h) (Table 2).

For adults, the dose calculation also considered the difference in factor values between agricultural workers and office workers due to different times spent indoors.

### Cohort members exposed in utero

The radiation dose to the thyroid gland of the foetus arising from ingestion by the mother of food products contaminated with ^131^I and short-lived radioiodine and radiotellurium isotopes (^132^Te+^132^I, ^133^I, and ^135^I) was calculated as:

20 $$\begin{aligned}\:{D}_{j,foetus}^{ing}=&\sum_{g={a}_{g,TOA}}^{{a}_{g,TOA+80}}{DC}_{j,foetus}^{ing}\left({a}_{g}\right)\\&\cdot\:\sum_{i}{A}_{j,i}^{milk,TIA}\cdot\:{PF}_{j,i}\cdot\:{V}_{i,preg}\:,\end{aligned}$$

where $\:{D}_{j,foetus}^{ing}$ is the dose to the thyroid gland of the foetus from ingestion of radionuclide *j* by the mother of a cohort member (Gy); $\:{DC}_{foetus}^{ing,j}\left({a}_{g}\right)\:$is the thyroid dose coefficient to a foetus of gestation age $\:{a}_{g}\:$from ingestion of radionuclide *j* by the mother (ICRP 2001) (Gy Bq^−1^); $\:{a}_{g,TOA}$ is the gestational age on the day of fallout (d);$\:\:{a}_{g,TOA+80}$ is the gestational age at 80 days after fallout when ^131^I decayed to a negligible level of 0.1% of the initial deposition (d); and $\:{V}_{i,preg}$ is the consumption rate of foodstuff *i* by the pregnant mother of a cohort member (L d^−1^).

Ethnicity-specific consumption rates of milk and dairy products by pregnant women were obtained during the focus groups study. Because it was not possible to collect information in 1998 on consumption (yes/no) and frequence of consumption during pregnancy for mothers of *in utero* exposed cohort members, the consumption rate of foodstuff *i* by pregnant women was calculated with Eq. 21:

21 $$\:{V}_{i,preg}={P}_{cons,preg}\cdot\:\:{V}_{i,preg}^{*},$$

where $\:{V}_{i,preg}$ is the average group-specific consumption rate of foodstuff *i* by the pregnant women included in the present study (L d^−1^) (Table 6); $\:{P}_{cons,preg}$ is the fraction of consumers of foodstuff *i* among pregnant women reported by focus groups (Drozdovitch et al. 2011) (unitless); and $\:{V}_{i,preg}^{*}$ is the consumption rate of foodstuff *i* by women as reported in the focus groups study who reported non-zero consumption during pregnancy (L d^−1^).

The thyroid dose to a foetus, $\:{D}_{j,foetus}^{inh}$, arising from inhalation of contaminated air by the mother was calculated as:

22 $$\:{D}_{j,foetus}^{inh}={D}_{j,mother}^{inh}\cdot\:{DC}_{j,foetus}^{inh}\left({a}_{g,TOA}\right)/{DC}_{j,mother}^{inh}\:,$$

where $\:{D}_{j,mother}^{inh}$ is the maternal thyroid dose from inhalation of radionuclide *j* by the mother calculated using Eq. (14) (Gy); $\:{DC}_{j,foetus}^{inh}\left({a}_{g,TOA}\right)\:$is the thyroid dose to a foetus of gestational age $\:{a}_{g,TOA}$ on the day of fallout deposition per unit of acute inhalation intake of radionuclide *j* by the mother (ICRP 2001) (Gy Bq^−1^); and $\:{DC}_{mother,j}^{inh}$ is the inhalation thyroid dose coefficient for radionuclide *j* for an adult individual given by Melo et al. (2022) (Gy Bq^−1^).

### Breastfed cohort members

The thyroid dose to a breastfed child from ingestion of radioiodine and radiotellurium isotopes by the mother was calculated as in Eq. 23:

23 $$\:{D}_{j,brfed}^{ing}={DC}_{j,brfed}^{ing}\cdot\:\sum_{i}{A}_{j,i}^{milk,TIA}\cdot\:{PF}_{j,i}\cdot\:{V}_{i,lact}\:\:,$$

where $\:{D}_{j,brfed}^{ing}$ is the thyroid dose to a breastfed child resulting from maternal ingestion of radionuclide *j* (Gy); $\:{DC}_{j,brfed}^{ing}\:$is the thyroid dose coefficient for a breastfed child from ingestion of radionuclide *j* by the mother (ICRP 2004) (Gy Bq^−1^); and $\:{V}_{i,lact}$ is the consumption rate of foodstuff *i* by lactating women (L d^−1^).

Ethnicity-specific consumption rates of milk and dairy products by lactating women were obtained during the focus groups study. By analogy with pregnant women, the consumption of foodstuff *i* by lactating women used in the present study was calculated from the focus groups data as (Eq. 24):

24 $$\:{V}_{i,lact}={P}_{cons,lact}\cdot\:{V}_{i,lact}^{*}\:,$$

where $\:{V}_{i,preg}$ is the average group-specific consumption rate of foodstuff *i* by lactating women used in this study (L d^−1^), given in Table 6; $\:{P}_{cons,lact}$ is the fraction of consumers of foodstuff *i* among lactating women reported by focus groups (unitless); and $\:{V}_{i,lact}^{*}$ is the consumption rate of foodstuff *i* by women from the focus groups study who reported non-zero consumption during lactation (L d^−1^).

According to a focus groups study, the typical duration of breastfeeding was 12 months. Supplemental foods other than breast milk, such as cow’s milk and sour milk, were introduced to infants (under 1 year of age), on average, at 6 months of age for 34% of Kazakh and at 8 months for 71% of Russian infants (Drozdovitch et al. 2011). The consumption rates of cow’s milk and sour milk by infants are given in Table 6. The thyroid dose to the infant due to his/her own consumption of supplemental foods was calculated using Eq. (5).

The thyroid dose to a breastfed child from inhalation of contaminated air was calculated using Eq. (14). In addition, the thyroid dose to a breastfed child due to inhalation of contaminated air by the mother was also calculated as (Eq. 25):

25 $$\:{D}_{j,brfed}^{inh}={C}_{j,mother}^{inh}\cdot\:{DC}_{j,brfed}^{inh}\:,$$

where $\:{D}_{j,brfed}^{inh}$ is the thyroid dose to a breastfed child due to inhalation of radionuclide *j* by the mother (Gy); $\:{C}_{j,mother}^{inh}$ is the activity of radionuclide *j* inhaled by the mother (Bq); and $\:{DC}_{j,brfed}^{inh}\:$is the thyroid dose to a breastfed child per unit of inhalation of radionuclide *j* by the mother (ICRP 2004) (Gy Bq^−1^).

### Evacuation of 1953

Residents of the villages of Karaul, Kengirbai, and Sarzhal were evacuated to the west in Karaganda Oblast in connection with the thermonuclear test #4 conducted on 12 August 1953. No fallout from the test was detected at any location where residents were resettled. Residents were allowed to return to Karaul and Kengirbai 10 days after the evacuation, and to Sarzhal 16 days after the evacuation.

According to Logachev (1997), 191 individuals did not manage to leave Karaul before the arrival of the radioactive cloud and were exposed to high levels of external irradiation. However, the cohort members living in Karaul at the time of the test were children, adolescents, and young adults aged up 25 y old. Since the youngest people were evacuated first, it is unlikely that anyone from the study cohort remained in Karaul when the radioactive cloud arrived and therefore received a high dose from external irradiation.

For residents of evacuated settlements, the integral of the exposure rate normalized to $\:\dot{X}\left(12\right)$ = 1 mR h^−1^ (see Eqs. (3) and (4) above) was calculated from the day the evacuees returned to their settlement, $\:{t}_return$, to the time of the end of residence in the settlement, *TC*, as (Eq. 26):

26 $$\:IF=\sum_{i=1}^{i=10}\frac{{a}_{i}}{{L}_{i}}\cdot\:\left({{e}^{{L}_{i}\cdot\:{t}_{return}}-e}^{{L}_{i}\cdot\:TC}\right)$$

where $\:{t}_{return}$ is the time from the day of the test until the day when the evacuees returned to their settlement of residence (d).

The time-integrated activity concentration of radioiodine and radiotellurium isotopes in milk for residents of evacuated settlements was calculated as (Eq. 27):

27 $$\begin{aligned}\:{A}_{j,i}^{milk,TIA}=\:&{A}_{j}^{grass}\left({t}_{TOA}\right)\cdot\:{I}_{a}\cdot\:{TF}_{a,j}\cdot\:\frac{{\lambda\:}_{b}^{j}}{\left({{\lambda\:}_{b}^{j}-\lambda\:}_{w}^{j}\right)}\\&\cdot\:{\int\:}_{{t}_{return}}^{TC}\left({e}^{-\left({\lambda\:}_{w}^{j}+{\lambda\:}_{r}^{j}\right)\cdot\:\tau\:}-{e}^{-\left({\lambda\:}_{b}^{j}+{\lambda\:}_{r}^{j}\right)\cdot\:\tau\:}\right)d\tau\:\end{aligned}$$

## Results and discussion

### Thyroid dose estimates

**Fig. 2:**
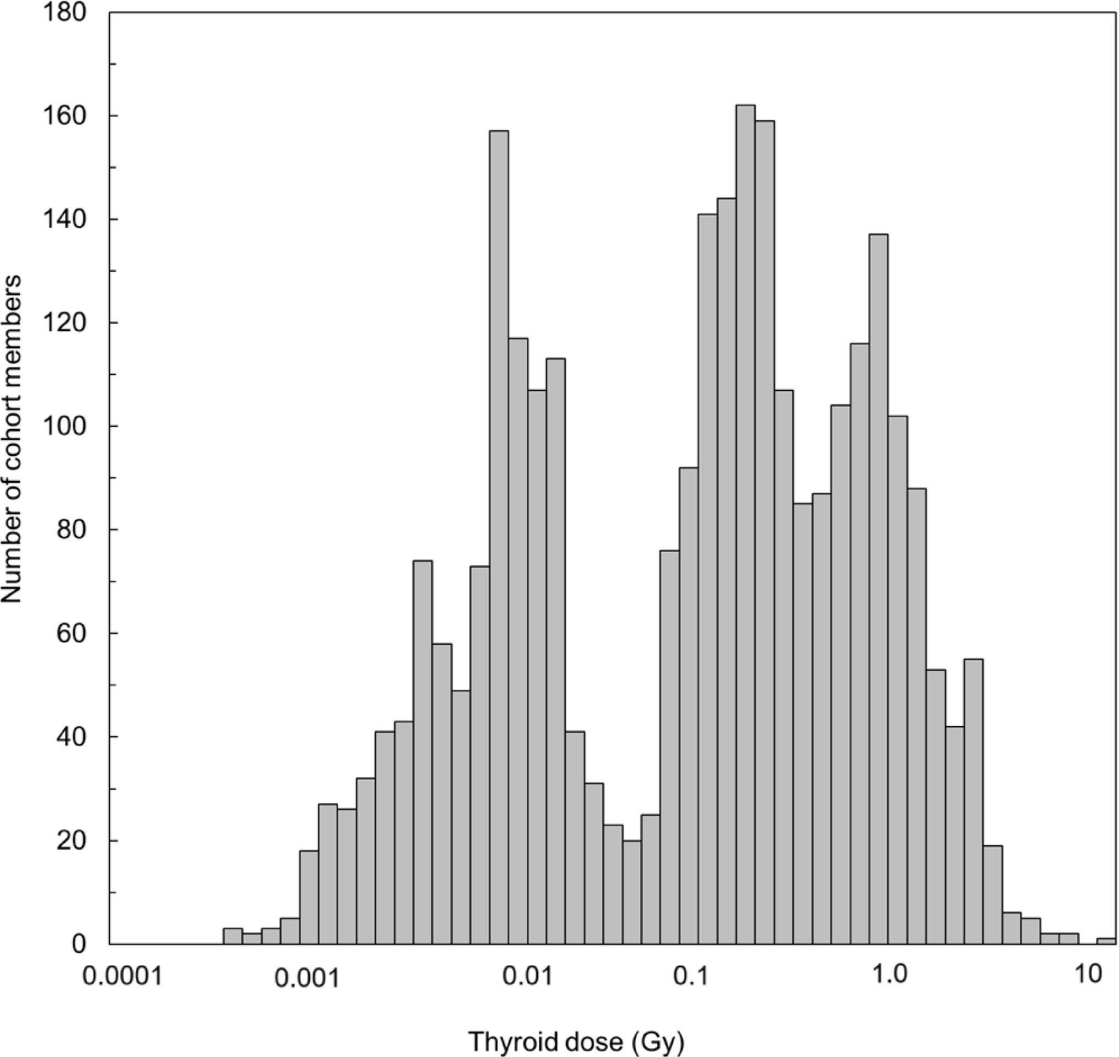
Distribution of total thyroid doses from all tests calculated for cohort members (logarithmic scale). Individuals with zero doses (*n*=309) are not shown

Figure 2 shows the distribution of the total dose to the thyroid from all tests. For 1,242 of 3,183 individuals (39.0% of the total), the thyroid dose from all tests varied between 0.1 Gy and 1.0 Gy while for 375 individuals (11.8%) the dose exceeded 1.0 Gy. The most exposed study participants, with individual thyroid dose higher than 1.0 Gy, mainly resided in the most affected settlements, including Dolon (*n* = 73 out of 108 individuals lived in the village) and Kanonerka (*n* = 113 out of 249) exposed primarily to fallout from test #1 conducted on 29 August 1949, and Abraly (*n* = 35 out of 69) and Kaynar (*n* = 128 out of 312) exposed primarily to fallout from test #2 conducted on 24 September 1951.

Individuals with zero doses (*n* = 309 out of 3,183, 9.7% of the total) are not shown in Fig. 2. Among them, 140 (4.4% of the total) individuals were not exposed to fallout, although they lived in the settlements affected by the test at some point between 1949 and 1962, because they either left the settlement before the test was conducted, or arrived or were born in the settlement much later after the test. Note that those 140 individuals were not exposed to fallout doses from inhalation or ingestion from short-lived radioiodine and radiotellurium isotopes, but it is likely that they were exposed to a very small dose from external irradiation due to the persistent soil contamination by long-lived gamma-emitting radionuclides such as ^137^Cs. That very small dose was ignored in the definition of the zero dose. The remaining 169 of the 309 unexposed individuals lived in 14 settlements that were not contaminated by any of the tests (Fig. 1).

Table 7 provides the distribution of thyroid doses from different exposure pathways in the cohort. Almost half of the cohort (1,566 out of 3,183; 49.2% of the total) received thyroid doses less than 0.1 Gy from all exposure pathways combined. A total of 732 individuals (23.0%) received thyroid doses of 0.5 Gy or higher, primarily from internal irradiation, as only 27 cohort members received thyroid doses of more than 0.5 Gy from external irradiation. Neither external irradiation nor inhalation of radioiodine and radiotellurium isotopes resulted in thyroid doses of more than 1 Gy. For the individual with the highest thyroid dose in the study (13.7 Gy), the doses from external irradiation, ingestion, and inhalation were 0.33 Gy, 13.3 Gy and 0.11 Gy, respectively.

**Table 7:** Distribution of thyroid dose from different exposure pathways in the cohort

Interval of thyroid dose (Gy)	External irradiation		Ingestion		Inhalation		Total	
	*N*	%	*N*	%	*N*	%	*N*	%
0	309^a^	9.7	372^b^	11.7	776^c^	24.4	309	9.7
0–0.0199	1,420	44.6	1,033	32.4	1,136	35.7	951	29.9
0.020–0.0499	500	15.7	260	8.2	316	9.9	112	3.5
0.050–0.0999	592	18.6	292	9.2	315	9.9	194	6.1
0.100–0.199	270	8.5	306	9.6	587	18.4	447	14.0
0.200–0.499	65	2.0	367	11.5	53	1.7	438	13.8
0.500–0.999	27	0.9	286	9.0	–	–	357	11.2
> 1.0	–	–	267	8.4	–	–	375	11.8
Entire cohort	3,183	100.0	3,183	100.0	3,183	100.0	3,183	100.0

^a^ The reasons for the zero dose (moving from or to a settlement before or after the affecting test, residence in non-contaminated settlement) are described in the text in detail

^b^ In addition to the 309 individuals with zero external dose, 63 cohort members lived in settlements affected by tests conducted in late October and March (##16, 18 and 26) when dairy animals were not on pasture and therefore the ingestion dose was zero

^c^ In addition to the 309 individuals with zero external dose, 467 cohort members lived in the villages of Karaul, Kengirbai, and Sarzhal evacuated for the thermonuclear test #4 conducted on 12 August 1953, and therefore their inhalation dose was zero

Table 8 shows the arithmetic mean (AM) of thyroid doses from all tests by age at first exposure and exposure pathway. The AM of thyroid doses from all exposure pathways was 0.43 Gy and the median was 0.13 Gy. As expected, the thyroid dose from internal irradiation decreased with increasing age. The estimated thyroid dose from external irradiation ranged from 4.9 × 10^−5^ Gy to 0.58 Gy (not shown) with an AM dose of 0.048 Gy and a median dose of 0.023 Gy. The thyroid dose from ingestion of ^131^I, ^132^Te+^132^I, ^133^I and ^135^I was from 0 to 13.3 Gy (AM: 0.34 Gy, median: 0.062 Gy), while the thyroid dose from inhalation ranged from 0 to 0.28 Gy (AM: 0.046 Gy, median: 2.8 × 10^−3^ Gy). A moderate correlation was found between individual external and internal thyroid doses, demonstrated by a Pearson correlation coefficient of $\:{r}_{p}$=0.35 (*p* < 0.001).

**Table 8:** Average thyroid dose from all tests by age at first exposure

Age at first exposure (y)	Number of persons	Average thyroid dose (Gy)			
		External	Internal		Total
			Ingestion	Inhalation	
*In utero*:	58				
Prenatal		0.065	0.98	0.053	1.1
Postnatal		3.5 × 10^−3^	9.3 × 10^−3^	0	0.013
0–4.99	668	0.048	0.58	0.056	0.69
5–9.99	632	0.045	0.32	0.052	0.41
10–14.99	798	0.053	0.28	0.049	0.38
15 +	718	0.046	0.13	0.028	0.21
Entire cohort^a^	2,874	0.048	0.34	0.046	0.43

^a^ 309 individuals with zero doses are excluded

Table 9 shows, as an example, the settlement-specific thyroid doses by exposure pathway from all tests received by cohort members resided in the selected settlements (listed in alphabetical order). The settlement-specific AM thyroid doses varied widely from several mGy to more than 1 Gy. The highest AM thyroid doses, 1 Gy and more, were estimated for the cohort members resided in the villages of Dolon and Kanonerka, with the major contribution from test #1, and in the villages of Abraly, Akbulak, and Kaynar, with the major contribution from test #2. Note that all cohort members from Abraly, Akbulak, and Kaynar (except one individual) were ethnic Kazakhs, and they reported consumption of mare milk and koumiss in 1949–1962. This resulted in significantly higher thyroid doses, because the concentration of radioiodine isotopes in mare milk was almost 8 times higher than in cow milk due to the significantly lower milk productivity of mares compared to cows (Simon S.L., personal communication, 2024) (Table 3).

**Table 9:** Settlement-specific thyroid doses from all considered tests received by cohort members resided in selected settlements

Settlement	Number of individuals	Thyroid dose from all considered tests (Gy) from					
		External irradiation		Internal irradiation^a^		Total	
		AM ± SD^b^	GM^c^	AM ± SD	GM	AM ± SD	GM
Abraly	69	0.036 ± 0.007	0.035	1.3 ± 1.1	0.90	1.3 ± 1.1	0.95
Akbulak	9	0.056 ± 0.010	0.056	1.2 ± 1.1	0.90	1.3 ± 1.1	0.97
Beskaragai	142	1.4 × 10^−3^ ± 3.1 × 10^−4^	1.4 × 10^−3^	0.017 ± 0.014	0.014	0.018 ± 0.014	0.015
Dolon	108	0.39 ± 0.20	0.13	1.2 ± 1.7	0.41	1.6 ± 1.7	0.61
Kanonerka	249	0.13 ± 0.048	0.081	1.0 ± 0.88	0.50	1.1 ± 0.90	0.61
Karaul^d^	446	0.058 ± 0.010	0.057	0.092 ± 0.10	0.055	0.15 ± 0.10	0.13
Kaynar	312	0.027 ± 0.006	0.025	1.2 ± 1.0	0.86	1.2 ± 1.0	0.89
Korostely	137	0.015 ± 0.006	9.5 × 10^−3^	0.30 ± 0.36	0.15	0.31 ± 0.36	0.16
Sarzhal^d^	202	0.090 ± 0.012	0.089	0.12 ± 0.053	0.11	0.21 ± 0.051	0.21
Zhurekadyr	14	0.021 ± 0.004	0.020	0.37 ± 0.23	0.29	0.39 ± 0.23	0.33

^a^ Sum of ingestion and inhalation doses

^b^ AM = arithmetic mean; SD = standard deviation

^c^ GM = geometric mean

^d^ Settlement was evacuated for the thermonuclear test #4 on 12 August 1953

Table 10 shows the characteristics of the total thyroid dose received by cohort members after each of the atmospheric nuclear weapons tests considered in the study. The highest thyroid doses were received after test #2 conducted on 24 September 1951 (AM ± standard deviation (AM ± SD) of doses was 1.1 ± 1.0 Gy, the geometric mean (GM) was 0.70 Gy), followed by test #1 conducted on 29 August 1949 (AM ± SD: 0.49 ± 0.90 Gy, GM: 0.047 Gy) and thermonuclear test #4 conducted on 12 August 1953 (AM ± SD: 0.16 ± 0.12 Gy, GM: 0.14 Gy). AM thyroid doses between 0.03 Gy and 0.1 Gy were realized after tests #20 (0.066 Gy), #28 (0.057 Gy), #18 (0.054 Gy), and #32 (0.047 Gy). For all other tests, the AM thyroid doses were less than 0.03 Gy.

**Table 10:** Characteristics of the thyroid dose received by cohort members after each of the atmospheric nuclear weapons tests considered in the present study

Test #	Test date (dd/mm/yyyy)	Number of persons exposed to given test^a^	Thyroid dose^b^ (Gy)	
			AM ± SD^c^	GM^d^
1	29/08/1949	1,190	0.49 ± 0.90	0.047
2	24/09/1951	433	1.1 ± 1.0	0.70
4	12/08/1953	749	0.16 ± 0.12	0.14
6	03/09/1953	7	0.029 ± 0.023	0.021
8	10/09/1953	7	0.026 ± 0.020	0.019
13	05/10/1954	329	6.7 × 10^−3^ ± 0.014	3.6 × 10^−3^
16	23/10/1954	104	1.6 × 10^−3^ ± 3.4 × 10^−4^	1.6 × 10^−3^
18	30/10/1954	242	0.054 ± 0.021	0.042
19	29/07/1955	993	5.5 × 10^−3^ ± 5.1 × 10^−3^	4.3 × 10^−3^
20	02/08/1955	16	0.066 ± 0.036	0.059
26	16/03/1956	21	0.011 ± 0.010	6.8 × 10^−3^
28	24/08/1956	24	0.057 ± 0.083	0.023
32	10/09/1956	6	0.047 ± 0.016	0.045
148	07/08/1962	1,534	6.3 × 10^−3^ ± 0.034	2.8 × 10^−3^
172	25/09/1962	17	8.2 × 10^−3^ ± 5.3 × 10^−3^	6.8 × 10^−3^

^a^ Because the same individual could be exposed to fallout after different tests, the total number of individuals exceeds the number of exposed cohort members, 2,874

^b^ The sum of doses from external and internal irradiation

^c^ AM = arithmetic mean; SD = standard deviation

^d^ GM = geometric mean

**Fig. 3:**
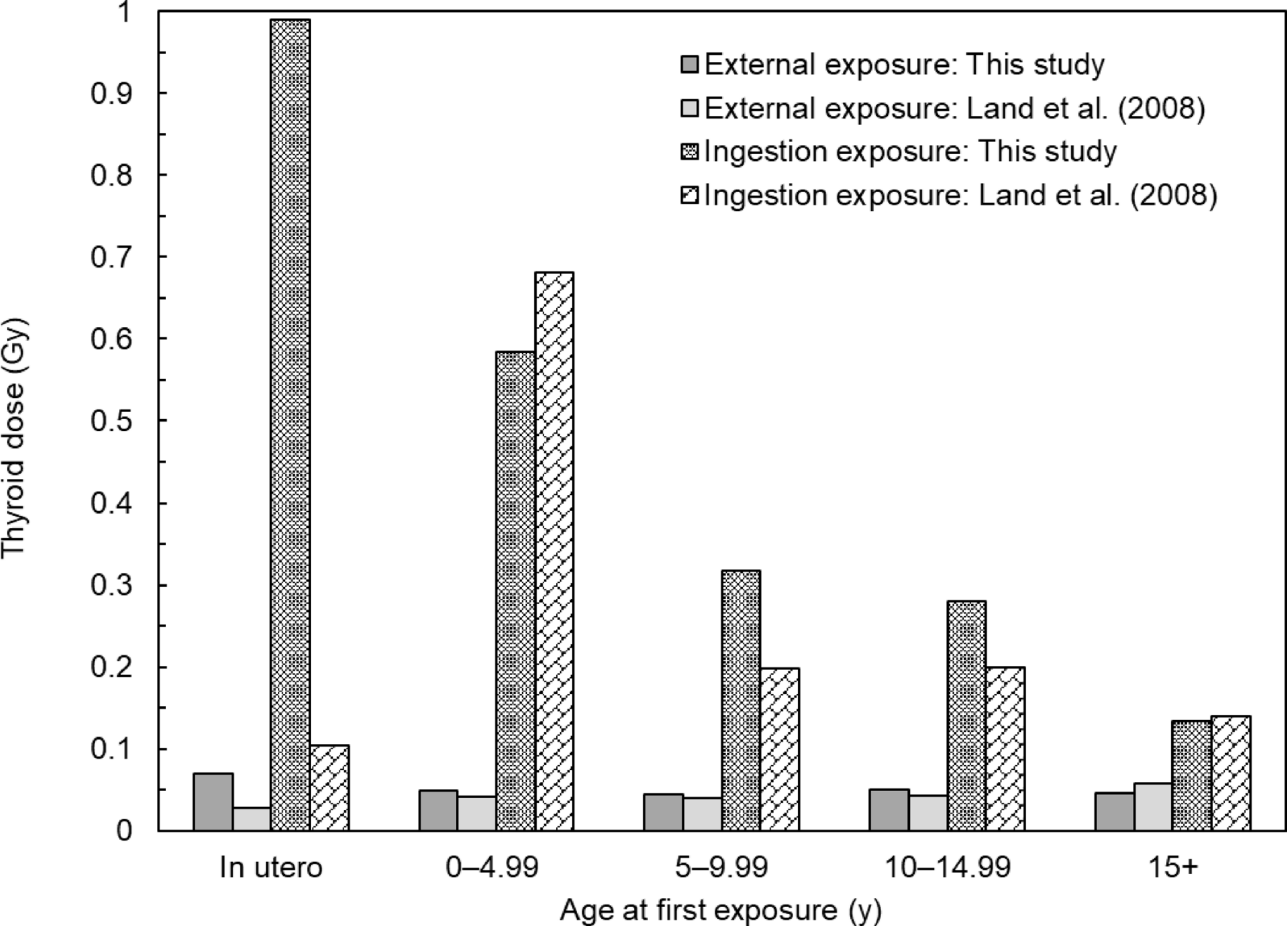
Comparison of arithmetic means of age-dependent thyroid doses calculated in the present study with those calculated by Land et al. (2008)

Figure 3 compares the age-dependent thyroid doses estimated in the present study with those calculated by Land et al. (2008). Because of changes in input data, i.e., $\:\dot{X}\left(12\right)$, residential history, and dietary data, there were differences in the age-specific thyroid doses between the two studies. The most significant difference was observed in individuals exposed *in utero*, i.e., the AM thyroid doses from ingestion were 0.98 Gy and 0.104 Gy in the present study and by Land et al. (2008), respectively. This difference is due to the diet of the pregnant women used in the present study and study by Land et al. (2008). According to the focus groups data, pregnant women of Kazakh ethnicity in 1949–1962 widely consumed fresh mare milk (the fraction of consumers was reported to be 69%) and koumiss (83%), while 100% of pregnant women of Russian ethnicity consumed almost 1 L of fresh cow milk daily (Drozdovitch et al. 2011). This high intake, which was not considered by Land et al. (2008), resulted in high doses to the foetus thyroid in the present study.

**Fig. 4:**
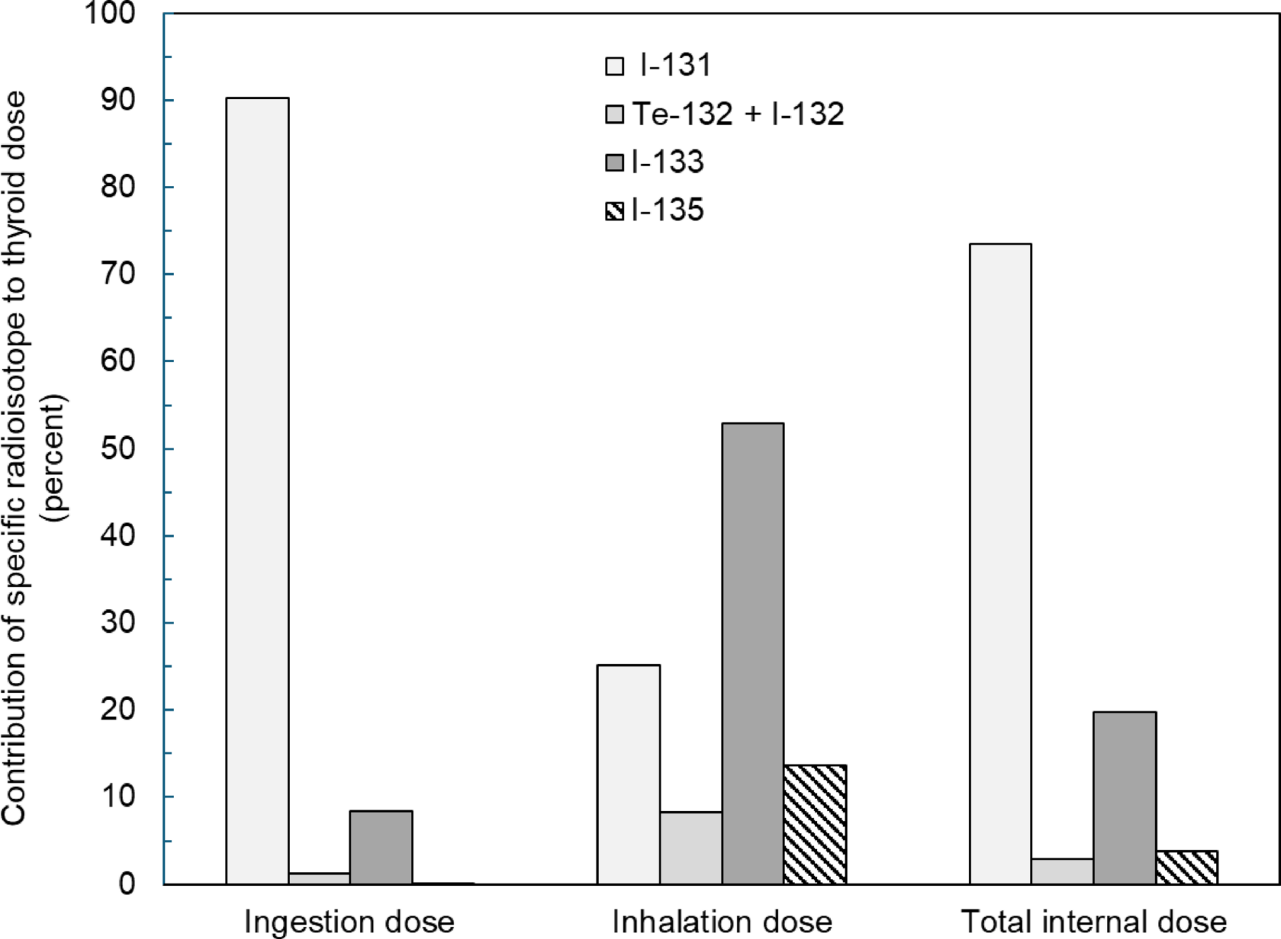
Contribution of radioiodine and radiotellurium isotopes to the thyroid doses from inhalation and ingestion exposure pathways and to the total internal thyroid dose in adult individuals of Russian ethnicity

Figure 4 shows the contribution of ^131^I, ^132^Te+^132^I, ^133^I and ^135^I to thyroid doses from inhalation and ingestion, as well as to the total internal thyroid dose in adult individuals of Russian ethnicity. More than 50% of the thyroid dose from inhalation was from ^133^I, followed by ^131^I (~ 25%), ^135^I (~ 14%), and ^132^Te+^132^I (~ 8%). Regarding the ingestion pathway of exposure due to consumption of cow milk and sour milk, the contribution of ^131^I to the thyroid dose was more than 90%, followed by ^133^I (~ 8.5%), and ^132^Te+^132^I (~ 1.3%), while the contribution of ^135^I, due to its short half-life of 6.6 h, was negligible (< 0.1%). For the total internal thyroid dose, ^131^I accounted for around 73%, followed by ^133^I (~ 20%), ^135^I (3.8%), and ^132^Te+^132^I (2.9%). For adult individuals of Kazakh ethnicity, ^131^I accounted for 82.5% of the total internal thyroid dose, followed by ^133^I (13.0%), ^132^Te+^132^I (2.3%), and ^135^I (2.2%).

Since the calculation of dose in the present study was based on the exposure rate at 12 h post-detonation, all deposited radionuclides contributing to external exposure, numbering about 150, were included in the dose assessment. The contributions of specific radionuclides to external exposure depends greatly on the TOA and the time interval of exposure. For example, the major contributors to external exposure in the period from 1 to 10 days after fallout with a TOA of 12 h are ^132^Te+^132^I (16%), ^140^Ba+^140^La (6.3%), and chain ^133m^Te→^133^Te→^133^I (4.1%), whereas for exposure from TOA of 12 h to 70 years after detonation the major contributors are ^132^Te+^132^I (21%), ^140^Ba+^140^La (21%), ^95^Zr+^95^Nb (7.6%), ^97^Zr+^97^Nb (7.5%), and ^103^Ru+^103^Rh (6.2%) (Bouville et al. 2022).

Although the same radionuclides are emitted in a nuclear detonation and in a reactor accident, their distributions are different, as the short-lived radionuclides are more prominent in a nuclear detonation. For example, the activity ratio of ^131^I to ^137^Cs is about 800 during the first few days after a nuclear detonation (Hicks 1981), while it was about 20 after the Chernobyl accident (Minenko et al. 2006).

### Comparison with doses obtained by EPR dosimetry

Table 11 compares the settlement-specific thyroid doses from external irradiation estimated in the present study for cohort members with dose estimates based on electron paramagnetic resonance (EPR) measurement in tooth enamel reported in the scientific literature (Ivannikov et al. 2002, 2006; Sholom et al. 2007; Zhumadilov et al. 2006, 2013). It is important to emphasize that the values of thyroid doses and doses to tooth enamel are not necessarily identical. However, they are very similar, given the close location of these two tissues in the human body and therefore they can be compared.

**Table 11:** Comparison of thyroid doses due to external irradiation estimated in the present study for cohort members with doses to tooth enamel measured using the electron paramagnetic resonance (EPR) method

Settlement	Number of cohort members	External dose estimated in the present study (Gy)		Number of teeth samples^a^	External dose estimated by EPR method (Gy)		References
		AM ± SD^b^	GM^c^		AM ± SD	GM	
Bodene	8	0.085 ± 0.027	0.079	6	0.12 ± 0.12 ^c^	0.076^d^	Zhumadilov et al. (2006)
Dolon	108	0.39 ± 0.20	0.13	14	0.18 ± 0.15 ^c^	0.13^d^	Ivannikov et al. (2002, 2006) Zhumadilov et al. (2006)
				13	0.22 ± 0.47	0.089^e^	Sholom et al. (2007)
Kanonerka	249	0.13 ± 0.048	0.081	7	0.23 ± 0.46	0.058^e^	Sholom et al. (2007)
Karaul	446	0.058 ± 0.010	0.057	12	0.13 ± 0.097	0.089^e^	Sholom et al. (2007)
Kaynar	312	0.027 ± 0.006	0.025	8	0.069 ± 0.12	0.032^e^	Sholom et al. (2007)
Kurchatov	7	0.010 ± 4.1 × 10^−4^	0.010	5	0.011 ± 0.020	–	Zhumadilov et al. (2013)
Mostik	5	0.064 ± 0.10	0.063	7	0.045 ± 0.041 ^c^	0.048^d, e^	Ivannikov et al. (2006)
Sarzhal	202	0.090 ± 0.012	0.089	12	0.16 ± 0.30	0.044^e^	Sholom et al. (2007)
Znamenka	5	0.031 ± 0.002	0.030	9	0.041 ± 0.046	–	Zhumadilov et al. (2013)

^a^ Tooth enamel formed before first exposure

^b^ AM = arithmetic mean; SD = standard deviation

^c^ GM = geometric mean

^d^ Calculated based on individual measurements provided in the references

^e^ Median

The two sets of doses show reasonable agreement: the AM ± SD of the ratios of the arithmetic mean of the settlement-specific doses estimated in the present study to the arithmetic mean of the EPR-based doses was 1.0 ± 0.61, while the GM of the ratios was 0.83; with a Pearson’s correlation coefficient of $\:{r}_{p}$=0.73 ($\:p$=0.01). The geometric mean of settlement-specific doses estimated in the present study correlated with the geometric mean of the EPR-based doses ($\:{r}_{p}$=0.83, $\:p$=0.006), while the ratio of geometric means of settlement-specific doses was characterized by an AM ± SD of 1.2 ± 0.44 and the GM of the ratios was 1.1. It should be emphasized, however, that the results of the comparison should be interpreted with the understanding that for most settlements the number of individuals for whom doses were assessed in the present study and by the EPR method was less than 10, and hence these individuals may not be a representative sample of the entire population of the settlement.

#### Uncertainties in doses

There are several major sources of uncertainties in thyroid doses estimated in the present study for the study participants as summarized below.

- Uncertainties, arising from the stochastic random variability of the parameters of the dose calculation methodology and from a lack of knowledge about the true values of the parameters, and errors in instrumental radiation measurement: Beck et al. (2022) indicated that the uncertainties and errors in $\:\dot{X}\left(12\right)$ values estimated from the measured exposure rate depend on several factors, including the measurement error, uncertainty in the decay correction from the time of measurement to H + 12, and the amount, type and quality of available data. Taking into account uncertainties in other parameters, Bouville et al. (2022) estimated that uncertainties in external doses due to radioactive fallout after atmospheric tests conducted at the Nevada Test Site are characterized by a geometric standard deviation (GSD), assuming a lognormal distribution, of 1.5–1.6, while significantly larger GSD values from 1.9 to 2.6 are typical for internal doses (Anspaugh et al. 2022). Of note, the uncertainties in doses are considered greater for SNTS tests, where there appears to be insufficient information on how and when exposure rate measurements were made.
- Reconstructed values of $\:\dot{X}\left(12\right)$ and TOA in 111 settlements not covered by measurements after atmospheric nuclear weapons tests conducted at the SNTS: Uncertainties in the $\:\dot{X}\left(12\right)$ values were caused by simplifying assumptions in the approximation approaches along and across the fallout trace that were used to reconstruct exposure rate values based on measurements done in neighboring settlements. For settlements where measurements were not performed, uncertainties in the estimated values of the exposure rate can reach factors 2.0 to 2.5 around the best estimate (Drozdovitch et al. 2025). As pointed out by Beck et al. (2022), if TOA were not based on actual exposure rate measurements, any significant wind shear with height could lead to errors in TOA values estimated using an average wind speed (Eq. (2).
- Reconstructed values of $\:{\dot{X}}_{axis}$ that resulted in the uncertainties in thyroid doses estimated from ingestion (Eqs. (8) and (9) and inhalation (Eqs. (9) and (14): The exact location of the fallout trace axis was unknown for all the tests considered except test #1, for which multiple exposure rate measurements were reported (Archive 1994; Loborev et al. 1994; Shoikhet et al. 1997; SRIRME 1998) showing that the fallout trace axis could be satisfactory described by a line passing near the villages Dolon – Topolka – Naumovka – Lokot. For all other tests considered in the present study, the results of the exposure rate measurements across the fallout trace axis, which were available, provided only an indirect indication of the trace axis location (Drozdovitch et al. 2025).
- Application of the methodology to wet deposited fallout after test #28: As discussed by Beck et al. (2022), “rain-out and wash-out will likely increase the probability of more of the smaller particles in the air and cloud being deposited at an earlier time, the average $\:R/V$ at the site will likely be reduced, and the fraction of total activity on less than 50 µm particles will likely be greater.” In the present study, it was considered that the fraction of radioactivity intercepted by vegetation during wet deposition was smaller than that for dry deposition (Eq. (13), and therefore this compensated to some extent for the possible overestimation of grass contamination in the event of wet deposition. Beck et al. (2022) concluded that although the methodology is primarily applicable only for dry deposition, it can also be used for wet deposition, but with higher uncertainty of the results.
- The state of iodine deficiency among inhabitants of the study area, determining the values of the thyroidal uptake and thyroid mass: In the present study, the thyroid dose coefficients calculated based on a thyroidal uptake of 30% were used, and the age-dependent values of the thyroid mass recommended by ICRP for stable iodine sufficient areas were taken from (ICRP 2002). It is challenging to estimate the degree of iodine deficiency around the time of tests in the 1949–1962 because data for this period are not available. However, an increase in the thyroid mass due to iodine deficiency would likely be compensated by an increase in the thyroid uptake (Zvonova 1989).
- Uncertainties associated with the information obtained in 1998, i.e., 36–49 years after the exposure to fallout in 1949–1962, during personal interviews on individual frequence of consumption of different types of milk and dairy products: It is generally recognized that recall of diet in distant past is strongly influenced by present dietary habits (Dwyer et al. 1989; Thompson et al. 1987) and is characterized by low reliability if the recall time exceeds 10 years (Willett 1998).

The uncertainties in the doses assessed in the present study were not quantified. Based on an extensive assessment of uncertainties in thyroid doses by Land et al. (2015) for a subset of the cohort, however, it was subjectively estimated that uncertainties in the present study are characterized by a GSD from 2.0 to 4.0 for most individuals.

### Strengths and limitations of the present study

The strengths of the present study are (i) the use of individual residential histories that have been verified for all cohort members, (ii) the use of individual questionnaire data on frequency of consumption of different types of milk and dairy products during the testing period, (iii) the availability of exposure rate values, measured or reconstructed, in all 146 affected settlements where the cohort members resided during the testing period in 1949–1962, and (iv) the doses from external irradiation estimated in this study for the cohort members are in reasonable agreement with doses estimated using EPR measurements in tooth enamel.

However, the present study has the following limitations:

- There is a lack of information on individual consumption of milk and dairy products during pregnancy and lactation for mothers of *in utero* exposed and breastfed cohort members. Although realistic consumption data were collected through a focus groups study, the consumption rates for pregnant and lactating women calculated using Eqs. (21) and (24) only represent average group-specific values.
- To the best of the authors’ knowledge, the U.S.-Russian methodology has never been used to calculate doses from inhalation in epidemiological studies among individuals exposed to fallout from atmospheric nuclear weapons tests. The authors believe that the application of the methodology for calculating thyroid doses from this exposure pathway requires careful consideration, because the dose estimates have never been validated through measurements or even calculations.

### Comparison with other exposed populations

Table 12 compares the thyroid doses calculated for the cohort in the present study with thyroid doses received by other populations exposed to fallout after the Chernobyl accident and atmospheric nuclear weapons tests conducted elsewhere. The study cohort received quite high doses compared to other exposed populations. For *in utero* exposed individuals, the thyroid doses from the Semipalatinsk NTS fallout (AM dose: 1.1 Gy; median dose: 0.27 Gy) were much higher than those from the Chernobyl fallout received in Belarus (AM dose: 0.12 Gy; median dose: 0.014 Gy (Drozdovitch et al. 2020), and Ukraine (AM dose: 0.083 Gy; median dose: 0.015 Gy (Masiuk et al. 2022), due to consumption of mare milk and koumiss by pregnant women in Kazakhstan. However, the number of *in utero* exposed individuals included in the present study cohort is small (*n* = 58).

**Table 12:** Comparison of the thyroid doses calculated for the study cohort with thyroid doses received by other populations exposed to fallout after the Chernobyl accident and atmospheric nuclear weapons tests conducted elsewhere

Nuclear test site/reactor accident	Period of exposure	Number of individuals	Age at exposure (y)	Thyroid dose^a^ (Gy)		References
				Mean	Median	
*Exposed in utero*						
Semipalatinsk	1949–1962	58	–	1.1	0.27	This study
Chernobyl (Belarus)	1986	2,965	–	0.12	0.014	Drozdovitch et al. (2020)
Chernobyl (Ukraine)	1986	2,582	–	0.083	0.015	Masiuk et al. (2022)
*Exposed as children and young adults*						
Semipalatinsk	1949–1962	2,816	< 36	0.42	0.13	This study
Nevada (Utah cohort)	1951–1962	2,497	0–18	0.12	0.055	Kerber et al. (1993), Simon et al. (2006b)
French Polynesia	1966–1974	804	< 34	4.7 × 10^−3^	3.7 × 10^−3^	Drozdovitch et al. (2021)
Chernobyl (Belarus)	1986	11,732	0–18	0.68	0.27	Drozdovitch et al. (2015)
Chernobyl (Ukraine)	1986	13,204	0–18	0.60	0.22	Masiuk et al. (2023)

^a^ The sum of doses from external and internal irradiation

Thyroid doses from the Semipalatinsk NTS fallout for individuals exposed as children and young adults (AM dose: 0.42 Gy; median dose: 0.13 Gy) show the same order of magnitude as doses received in the Belarusian-American (AM dose: 0.68 Gy, median dose: 0.27 Gy) and Ukrainian-American (AM dose: 0.60 Gy, median dose: 0.22 Gy) cohorts of individuals exposed to the Chernobyl fallout (Drozdovitch et al. 2015; Masiuk et al. 2023). The present cohort of individuals exposed *in utero* and at childhood and adolescence to high doses of radiation provides unique opportunities to assess radiation-related risks of thyroid cancer, thyroid nodules, and other structural and functional thyroid diseases.

## Concluding remarks

This paper presents estimates of thyroid doses for a cohort of 3,183 individuals who were exposed to fallout from atmospheric nuclear weapons tests conducted at the SNTS between 1949 and 1962. Individual thyroid doses due external irradiation from gamma-emitted radionuclides deposited on the ground and due to intake of ^131^I and of short-lived ^132^Te+^132^I, ^133^I, and ^135^I via (i) ingestion with locally produced cow and mare milk and dairy products and (ii) inhalation of contaminated air during the passage of the radioactive cloud have been reconstructed. The thyroid doses in the cohort were found to be quite high, i.e., the arithmetic mean of thyroid doses from all exposure pathways was 0.43 Gy (range from 3.5 × 10^−4^ Gy to 13.7 Gy) and the median was 0.13 Gy. Although the uncertainties in the doses, which were estimated in the present study, were not evaluated in a quantitative manner, they were subjectively estimated to be relatively high and to be characterized, on average, by a GSD from 2.0 to 4.0 for most individuals. The study cohort received high doses compared to other populations exposed to fallout from the Chernobyl accident and atmospheric nuclear weapons tests conducted elsewhere. The results of the present thyroid dose assessment are currently being used to evaluate the risk of thyroid nodules, thyroid cancer and other thyroid diseases in the study cohort.

## Data Availability

Access to the data for using by Third Party is limited to qualified investigators and will be granted to them under collaborative agreement with the Scientific Research Institute of Radiation Medicine and Ecology of the Semey Medical University (Semey, Republic of Kazakhstan).

## Footnotes

1 An oblast is the largest administrative unit in Republic of Kazakhstan. The typical size of an oblast in Kazakhstan is 100,000-200,000 km^2^ with a population of 0.5–1.0 million individuals. Oblasts are sub-divided into raions; there are typically about 10–15 raions of similar size and population in one oblast.

2 Names of administrative units, oblast or raion, are given as of January 2025. Abai Oblasts is the former Semipalatinsk Oblast.

3 The doses due to the consumption of breast milk are treated in the section “Breastfed cohort members” of this paper.

## Declarations

### **Disclaimer**

The contributions of the NIH authors were made as part of their official duties as NIH federal employees, are in compliance with agency policy requirements, and are considered Works of the United States Government. However, the findings and conclusions presented in this paper are those of the authors and do not necessarily reflect the views of the NIH or the U.S. Department of Health and Human Services. Where authors are identified as personnel of the International Agency for Research on Cancer/World Health Organization, the authors alone are responsible for the views expressed in this article and they do not necessarily represent the decisions, policy, or views of the International Agency for Research on Cancer/World Health Organization.

## References

[CR1] Anspaugh LR, Bouville A, Thiessen KM, Hoffman FO, Beck HL, Gordeev KI, Simon SL (2022) A methodology for calculation of internal dose following exposure to radioactive fallout from the detonation of a nuclear fission device. Health Phys 122:84–124. 10.1097/HP.0000000000001503 34898517 10.1097/HP.0000000000001503 PMC8677618

[CR2] Apsalikov KN, Lipikhina A, Grosche B, Belikhina T, Ostroumova E, Shinkarev S, Stepanenko V, Muldagaliev T, Yoshinaga S, Zhunussova T, Hoshi M, Katayama H, Lackland DT, Simon SL, Kesminiene A (2019) The state scientific automated medical Registry, Kazakhstan: an important resource for low-dose radiation health research. Radiat Environ Biophys 58(1):1–11. 10.1007/s00411-018-0762-5 30446811 10.1007/s00411-018-0762-5

[CR3] Archive (1994) From the report on measuring of the trace on the radioactive fallout (on P-2 in 1949). Bulletin of the scientific program semipalatinsk test site –. Altai 4:87–92 (in Russian)

[CR4] Beck HL, Bouville A, Simon SL, Anspaugh LR, Thiessen KM, Shinkarev S, Gordeev K (2022) A method for estimating the deposition density of fallout on the ground and on vegetation from a low-yield, low-altitude nuclear detonation. Health Phys 122:21–53. 10.1097/HP.0000000000001496 34898515 10.1097/HP.0000000000001496 PMC8677616

[CR5] Bouville A, Beck HL, Thiessen KM, Hoffman FO, Potischman N, Simon SL (2020) The methodology used to assess doses from the first nuclear weapons test (Trinity) to the populations of New Mexico. Health Phys 119:400–427. 10.1097/HP.0000000000001331 32881739 10.1097/HP.0000000000001331 PMC7497484

[CR6] Bouville A, Beck HL, Anspaugh LR, Gordeev K, Shinkarev S, Thiessen KM, Hoffman FO, Simon SL (2022) A methodology for estimating external doses to individuals and populations exposed to radioactive fallout from nuclear detonations. Health Phys 122:54–83. 10.1097/HP.0000000000001504 34898516 10.1097/HP.0000000000001504 PMC8677613

[CR7] Cahoon EK, Grimm E, Mabuchi K, Mai JZ, Zhang R, Drozdovitch V, Hatch M, Little MP, Peters KO, Bogdanova TI, Shelkovoy E, Shpak VM, Terekhova G, Zamotayeva G, Pasteur IP, Masiuk SV, Chepurny M, Zablotska LB, McConnell R, O’Kane P, Tronko MD, Brenner AV (2024) Prevalence of thyroid nodules in residents of Ukraine exposed as children or adolescents to Iodine-131 from the chornobyl accident. Thyroid 34:890–898. 10.1089/thy.2023.0654 38757581 10.1089/thy.2023.0654 PMC11295839

[CR8] de Vathaire F, Zidane M, Xhaard C, Souchard V, Chevillard S, Ory C, Rachédi F, Nunez S, Leufroy A, Noël L, Guérin T, Shan L, Bost-Bezeaud F, Petitdier P, Soubiran G, Allodji R, Ren Y, Doyon F, Taquet M, Gardon J, Bouville A, Drozdovitch V (2023) Assessment of differentiated thyroid carcinomas in French Polynesia after atmospheric nuclear tests performed by France. JAMA Netw Open 6:e2311908. 10.1001/jamanetworkopen.2023.11908 37145599 10.1001/jamanetworkopen.2023.11908 PMC10163383

[CR9] Doobasov UV, Zelentsov SA, Krasilov GA, Logachev VA, Matushenko AM, Smagoolov SG, Tsatoorov BS, Tsirkov GA, Chernishev AK (1994) Chronological list of the atmospheric nuclear tests at the semipalatinsk test site and their radiological characteristics. Bulletin of the scientific program semipalatinsk test site –. Altai 4:78–86 (in Russian)

[CR10] Drozdovitch V, Schonfeld S, Akimzhanov K, Aldyngurov D, Land CE, Luckyanov N, Mabuchi K, Potischman N, Schwerin MJ, Semenova Y, Tokaeva A, Zhumadilov Z, Bouville A, Simon SL (2011) Behavior and food consumption pattern of the population exposed in 1949–1962 to fallout from semipalatinsk nuclear test site in Kazakhstan. Radiat Environ Biophys 50:91–103. 10.1007/s00411-010-0334-9 20938673 10.1007/s00411-010-0334-9 PMC3853382

[CR11] Drozdovitch V, Minenko V, Golovanov I, Khrutchinsky A, Kukhta T, Kutsen S, Luckyanov N, Ostroumova E, Trofimik S, Voillequé P, Simon SL, Bouville A (2015) Thyroid dose estimates for a cohort of Belarusian children exposed to (131)I from the Chernobyl accident: assessment of uncertainties. Radiat Res 184:203–218. 10.1667/rr13791.1 26207684 10.1667/rr13791.1 PMC4548301

[CR12] Drozdovitch V, Minenko V, Kukhta T, Trofimik S, Grakovitch R, Hatch M, Cahoon EK, Veyalkin I, Polyanskaya O, Yauseyenka V, Ostroumova E, Mabuchi K, Rozhko A (2020) Thyroid dose estimates for a cohort of Belarusian persons exposed in utero and during early life to Chernobyl fallout. Health Phys 118:170–184. 10.1097/HP.0000000000001135 31869316 10.1097/HP.0000000000001135 PMC6931907

[CR13] Drozdovitch V, Bouville A, Taquet M, Gardon J, Xhaard C, Ren Y, Doyon F, de Vathaire F (2021) Thyroid doses to French Polynesians resulting from atmospheric nuclear weapons tests: estimates based on radiation measurements and population lifestyle data. Health Phys 120:34–55. 10.1097/HP.0000000000001262 33002966 10.1097/HP.0000000000001262 PMC7710602

[CR14] Drozdovitch V, Lipikhina A, Apsalikov K, Brait Y, Tokanov A, Yassilkanov G, Rosenson R, Ostroumova E (2025) Assessment of radiation contamination of villages in Northeastern Kazakhstan not covered by exposure rate measurements after atmospheric nuclear weapons tests conducted at the semipalatinsk nuclear test site. J Environ Radioact 288:107731. 10.1016/j.jenvrad.2025.107731 40499259 10.1016/j.jenvrad.2025.107731 PMC12220931

[CR15] Dwyer JT, Gardner J, Halvorsen K, Krall EA, Cohen A, Valadian I (1989) Memory of food intake in the distant past. Am J Epidemiol 130:1033–1046. 10.1093/oxfordjournals.aje.a115404 2816890 10.1093/oxfordjournals.aje.a115404

[CR16] Eckerman K, Endo A (2008) ICRP publication 107. Nuclear decay data for dosimetric calculations. Ann ICRP 38(3):7–96. 10.1016/j.icrp.2008.10.004 19285593 10.1016/j.icrp.2008.10.004

[CR17] Fesenko S, Howard BJ, Isamov N, Voigt G, Beresford NA, Sanzharova N, Barnett CL (2007) Review of Russian Language studies on radionuclide behavior in agricultural animals: part 2. Transfer to milk. J Environ Radioact 98:104–136. 10.1016/j.jenvrad.200706.007 17766017 10.1016/j.jenvrad.2007.06.007

[CR18] Gordeev KI (1999) Radiation exposure to the population of the Semipalatinsk region from Semipalatinsk weapons tests. Part I: experimental and theoretical investigation of the processes of radioactive contamination of grass resulting from local fallout from nuclear explosions and justification of the concept of biologically active fraction of fallout. Report to the National Cancer Institute #263–MQ–920145. Bethesda, MD, USA

[CR19] Gordeev KI (2001a) Radiation exposure to the population of the semipalatinsk region from semipalatinsk weapons tests. Part IV. Assessment of the realistic doses to whole-body from external gamma irradiation and doses to thyroid from internal irradiation for the populations living in the number of settlements of Kazakhstan as a result of radiation exposure from nuclear explosions conducted at the semipalatinsk polygon. Report to the National Cancer Institute #263–MQ–920145, Bethesda, MD, USA

[CR20] Gordeev KI (2001b) Radiation exposure to the population of the Semipalatinsk region from Semipalatinsk weapons tests. Part IV-2. Assessment of external effective doses and internal absorbed doses to thyroid for the population living in additional settlements of the Republic of Kazakhstan and of the Russian Federation, included by the NCI in the list of the cohort subjects for the epidemiological study of the medical effects due to radiation exposure as a result of nuclear testing at the Semipalatinsk Polygon. Final report to the National Cancer Institute #263–MQ–920145. Bethesda, MD, USA

[CR21] Gordeev KI, Lebedev AN, Savkin MN (1994) Method of retrospective reconstruction of radiological situation determining the internal irradiation upon the trace of nuclear test. Bulletin of the scientific program semipalatinsk test site –. Altai 1:57–96 (in Russian)

[CR22] Gordeev K, Vasilenko I, Lebedev A, Bouville A, Luckyanov N, Simon SL, Stepanov Y, Shinkarev S, Anspaugh L (2002) Fallout from nuclear tests: dosimetry in Kazakhstan. Radiat Environ Biophys 41:61–67. 10.1007/s00411-001-0139-y 12014413 10.1007/s00411-001-0139-y

[CR23] Gordeev K, Shinkarev S, Ilyin L, Bouville A, Hoshi M, Luckyanov N, Simon SL (2006a) Retrospective dose assessment for the population living in areas of local fallout from the semipalatinsk nuclear test site part I: external exposure. J Radiat Res 47(Suppl A):A129–136. 10.1269/jrr.47.a129 16571927 10.1269/jrr.47.a129

[CR24] Gordeev K, Shinkarev S, Ilyin L, Bouville A, Hoshi M, Luckyanov N, Simon SL (2006b) Retrospective dose assessment for the population living in areas of local fallout from the semipalatinsk nuclear test site part II: internal exposure to thyroid. J Radiat Res 47(Suppl A):A137–141. 10.1269/jrr.47.a137 16571928 10.1269/jrr.47.a137

[CR25] Hatch M, Brenner AV, Cahoon EK, Drozdovitch V, Little MP, Bogdanova T, Shpak V, Bolshova E, Zamotayeva G, Terekhova G, Shelkovoy E, Klochkova V, Mabuchi K, Tronko M (2019) Thyroid cancer and benign nodules after exposure *in utero* to fallout from Chernobyl. J Clin Endocrinol Metab 104:41–48. 10.1210/jc.2018-00847 30445441 10.1210/jc.2018-00847 PMC6456983

[CR26] Henderson RW (1991) Approximation of the decay of fission and activation product mixtures. Report LA-11968-MS. Los Alamos, NM: Los Alamos National Laboratory. https://inis.iaea.org/collection/NCLCollectionStore/_Public/22/044/22044664.pdf (last access on 3 January 2025)

[CR27] Hicks HG (1981) Results of calculations of external radiation exposure rates from fallout and the related radionuclides composition. LLNL Report UCRL-53152, Parts 3–4, 6–8 Livermore, CA

[CR28] Hoffman FO, Thiessen KM, Frank ML, Blaylock BG (1992) Quantification of the interception and initial retention of radioactive contaminants deposited on pasture grass by simulated rain. Atmos Environ. 10.1016/0960-1686(92)90348-O

[CR29] ICRP – International Commission on Radiological Protection (2001) Doses to the embryo and fetus from intakes of radionuclides by the mother. ICRP publication 88. Ann ICRP 31(1–3):19–515. 10.1016/S0146-6453(01)00022-7 11730884 10.1016/S0146-6453(01)00022-7

[CR30] ICRP – International Commission on Radiological Protection (2002) Basic anatomical and physiological data for use in radiological protection: reference values. ICRP publication 89. Ann ICRP 32(3/4):5–265. 10.1016/S0146-6453(03)00002-2 14506981

[CR31] ICRP – International Commission on Radiological Protection (2004) Doses to infants from ingestion of radionuclides in mothers’ milk. ICRP publication 95. Ann ICRP 34(3–4):15–280. 10.1016/j.icrp.2004.12.002 10.1016/j.icrp.2004.12.002 16168243

[CR32] Ivannikov AI, Zhumadilov Z, Gusev BI, Miyazawa C, Jiao L, Skvortsov VG, Stepanenko VF, Takada J, Hoshi M (2002) Individual dose reconstruction among residents living in the vicinity of the Semipalatinsk nuclear test site using EPR spectroscopy of tooth enamel. Health Phys 83:183–196. 10.1097/00004032-200208000-00004 12132707 10.1097/00004032-200208000-00004

[CR33] Ivannikov A, Zhumadilov K, Tieliewuhan E, Jiao L, Zharlyganova D, Apsalikov KN, Berekenova G, Zhumadilov Z, Toyoda S, Miyazawa C, Skvortsov V, Stepanenko V, Endo S, Tanaka K, Hoshi M (2006) Results of EPR dosimetry for population in the vicinity of the most contaminating radioactive fallout trace after the first nuclear test in the semipalatinsk test site. J Radiat Res 47(Suppl A):A39–46. 10.1269/jrr.47.a39 16571943 10.1269/jrr.47.a39

[CR34] Kerber RA, Till JE, Simon SL, Lyon JL, Thomas DC, Preston-Martin S, Rallison ML, Lloyd RD, Stevens W (1993) A cohort study of thyroid disease in relation to fallout from nuclear weapons testing. JAMA 270:2076–2082. 10.1001/jama.1993.03510170066032 8411574

[CR35] Land CE, Zhumadilov Z, Gusev BI, Hartshorne MH, Wiest PW, Woodward PW, Crooks LA, Luckyanov NK, Fillmore CM, Carr Z, Abisheva G, Beck HL, Bouville A, Langer J, Weinstock R, Gordeev KI, Shinkarev S, Simon SL (2008) Ultrasound-detected thyroid nodule prevalence and radiation dose from fallout. Radiat Res 169:373–383. 10.1667/RR1063.1 18363427 10.1667/RR1063.1 PMC4018569

[CR36] Land CE, Kwon D, Hoffman FO, Moroz B, Drozdovitch V, Bouville A, Beck H, Luckyanov N, Weinstock RM, Simon SL (2015) Accounting for shared and unshared dosimetric uncertainties in the dose response for ultrasound-detected thyroid nodules after exposure to radioactive fallout. Radiat Res 183:159–173. 10.1667/RR13794.1 25574587 10.1667/RR13794.1 PMC4423551

[CR37] Loborev VM, Sudakov VV, Zelenov VI, Gabbasov MN, Markovetsev AS, Djatchenko VI, Volobuev NM (1994) The reconstruction of Altai region population irradiation doses due to nuclear explosion of August 29, 1949. Bulletin of the scientific program semipalatinsk test site –. Altai 1:27–56 (in Russian)

[CR38] Loborev VM, Shoikhet YN, Sudakov VV, Zelenov VI, Gabbasov MN (1997) Doses of residents of the cities of semipalatinsk, Ust-Kamenogorsk, Kurchatov and the settlement of Chagan delivered by the nuclear tests at the semipalatinsk test site. Bulletin of the scientific program semipalatinsk test site –. Altai 1:51–64 (in Russian)

[CR39] Logachev VA (1997) Semipalatinsk test site: Ensuring general and radiation safety of nuclear tests. Moscow: IGEM RAN, 345 p. (In Russian). https://elib.biblioatom.ru/text/semipalatinskiy-poligon_1997/p345/ (last access on 3 January 2025)

[CR40] Lyon JL, Alder SC, Stone MB, Scholl A, Reading JC, Holubkov R, Sheng X, White GL Jr, Hegmann KT, Anspaugh L, Hoffman FO, Simon SL, Thomas B, Carroll R, Meikle AW (2006) Thyroid disease associated with exposure to the Nevada nuclear weapons test site radiation: a re-evaluation based on corrected dosimetry and examination data. Epidemiology 17:604–614. 10.1097/01.ede.0000240540.79983.7f 17028502 10.1097/01.ede.0000240540.79983.7f

[CR41] Masiuk S, Chepurny M, Buderatska V, Ivanova O, Boiko Z, Zhadan N, Hatch M, Cahoon EK, Zamotayeva G, Shpak V, Tronko M, Drozdovitch V (2022) Assessment of internal exposure to ^131^I and short-lived radioiodine isotopes and associated uncertainties in the Ukrainian cohort of persons exposed in utero. J Radiat Res 63(3):364–377. 10.1093/jrr/rrac007 35301522 10.1093/jrr/rrac007 PMC9124623

[CR42] Masiuk S, Chepurny M, Buderatska V, Ivanova O, Boiko Z, Zhadan N, Mabuchi K, Cahoon EK, Little MP, Kukush A, Bogdanova T, Shpak V, Zamotayeva G, Tronko M, Drozdovitch V (2023) Exposure to the thyroid from intake of radioiodine isotopes after the chornobyl accident. Report I: revised doses and associated uncertainties for the Ukrainian-American cohort. Radiat Res 199:61–73. 10.1667/RADE-21-00152.1 36366807 10.1667/RADE-21-00152.1 PMC9899004

[CR43] Melo DR, Bertelli L, Ibrahim SA, Anspaugh LR, Bouville A, Simon SL (2022) Dose coefficients for internal dose assessments for exposure to radioactive fallout. Health Phys 122:125–235. 10.1097/HP.0000000000001500 34898518 10.1097/HP.0000000000001500 PMC8677615

[CR44] MHRF – Ministry of Health of the Russian Federation (2001) Assessment of absorbed and effective doses of ionizing radiation in the population permanently living on radioactive traces of atmospheric nuclear tests. Methodical Guidelines MU 2.6.1.1001–00. Moscow: MHRF (In Russian)

[CR45] Minenko VF, Ulanovsky AV, Drozdovitch VV, Shemiakina EV, Gavrilin YI, Khrouch VT, Shinkarev SM, Voillequé PG, Bouville A, Anspaugh LR, Luckyanov N (2006) Individual thyroid dose estimates for a case-control study of Chernobyl-related thyroid cancer among children of Belarus – Part II. Contributions from long-lived radionuclides and external radiation. Health Phys 90:312–327. 10.1097/01.HP.0000183761.30158.c1 16538137 10.1097/01.HP.0000183761.30158.c1

[CR46] Müller H, Pröhl G (1993) ECOSYS-87: a dynamic model for assessing radiological consequences of nuclear accidents. Health Phys 64:232–252. 10.1097/00004032-199303000-00002 8432643 10.1097/00004032-199303000-00002

[CR47] NCRP – National Council on Radiation Protection and Measurements (1996) Screening models for releases of radionuclides to atmosphere, surface water, and ground. National Council on Radiation Protection and Measurements, Bethesda, MD. NCRP Report No. 123

[CR48] Ostroumova E, Brenner A, Oliynyk V, McConnell R, Robbins J, Terekhova G, Zablotska L, Likhtarev I, Bouville A, Shpak V, Markov V, Masnyk I, Ron E, Tronko M, Hatch M (2009) Subclinical hypothyroidism after radioiodine exposure: Ukrainian-American cohort study of thyroid cancer and other thyroid diseases after the Chornobyl accident (1998–2000). Environ Health Perspect 117:745–750. 10.1289/ehp.0800184 19479016 10.1289/ehp.0800184 PMC2685836

[CR49] Pröhl G (2009) Interception of dry and wet deposited radionuclides by vegetation. J Environ Radioact 100:675–682. 10.1016/j.jenvrad.2008.10.006 19027204 10.1016/j.jenvrad.2008.10.006

[CR50] Schwerin M, Schonfeld S, Drozdovitch V, Akimzhanov K, Aldyngurov D, Bouville A, Land C, Luckyanov N, Mabuchi K, Semenova Y, Simon S, Tokaeva A, Zhumadilov Z, Potischman N (2010) The utility of focus group interviews to capture dietary consumption data in the distant past: dairy consumption in Kazakhstan villages 50 years ago. J Dev Orig Health Dis 1:192–202. 10.1017/S2040174410000243 24286002 10.1017/S2040174410000243 PMC3839237

[CR51] Shoikhet YN, Kiselev VI, Loborev VM, Sudakov VV, Algazin AI, Demin VF, Lagutin AA (1997) Nuclear test of 29 August 1959. Radiation influence of population of Altai Krai. Nexi, Barnaul, p 267. (in Russian)

[CR52] Shoikhet Y, Loborev V, Sudakov V, Kiselev VI, Zelenov V, Azarov L (2002) Fallout from nuclear tests: dosimetry in the Altai region. Radiat Environ Biophys 41:57–60. 10.1007/s00411-001-0138-z 12014412 10.1007/s00411-001-0138-z

[CR53] Sholom S, Desrosiers M, Bouville A, Luckyanov N, Chumak V, Simon SL (2007) EPR tooth dosimetry of SNTS area inhabitants. Radiat Meas 42:1037–1040. 10.1016/j.radmeas.2007.05.007 19590746 10.1016/j.radmeas.2007.05.007 PMC2707028

[CR54] Simon SL, Beck HL, Gordeev K, Bouville A, Anspaugh LR, Land CE, Luckyanov N, Shinkarev S (2006a) External dose estimates for Dolon village: application of the U.S./Russian joint methodology. J Radiat Res 47(Suppl A):A143–147. 10.1269/jrr.47.a143 16571929 10.1269/jrr.47.a143

[CR55] Simon SL, Anspaugh LR, Hoffman FO, Scholl AE, Stone MB, Thomas BA, Lyon JL (2006b) 2004 update of dosimetry for the Utah thyroid cohort study. Radiat Res 165:208–222. 10.1667/rr3483.1 16435919 10.1667/rr3483.1

[CR56] Simon SL, Bouville A, Beck HL, Anspaugh LR, Thiessen KM, Hoffman FO, Shinkarev S (2022) Dose estimation for exposure to radioactive fallout from nuclear detonations. Health Phys 122:1–20. 10.1097/HP.0000000000001501 34898514 10.1097/HP.0000000000001501 PMC8677604

[CR57] SRIRME – Scientific Research Institute of Radiation Medicine and Ecology (1998) Archival data on settlements affected by atmospheric nuclear weapons tests conducted at the semipalatinsk nuclear test site. SRIRME, Semey. (in Russian)

[CR58] Thiessen KM, Hoffman FO, Bouville A, Anspaugh LR, Beck HL, Simon SL (2022) Parameter values for estimation of internal doses from ingestion of radioactive fallout from nuclear detonations. Health Phys 122:236–268. 10.1097/HP.0000000000001493 34898519 10.1097/HP.0000000000001493 PMC8677614

[CR59] Thompson FE, Lamphiear DE, Metzner HL, Hawthorne VM, Oh MS (1987) Reproducibility of reports of frequency of food use in the Tecumseh diet methodology study. Am J Epidemiol 125:658–671. 10.1093/oxfordjournals.aje.a114579 3826044 10.1093/oxfordjournals.aje.a114579

[CR60] Tronko M, Brenner AV, Bogdanova T, Shpak V, Oliynyk V, Cahoon EK, Drozdovitch V, Little MP, Tereshchenko V, Zamotayeva G, Terekhova G, Zurnadzhi L, Hatch M, Mabuchi K (2017) Thyroid neoplasia risk is increased nearly 30 years after the Chernobyl accident. Int J Cancer 141(8):1585–1588. 10.1002/ijc.30857 28662277 10.1002/ijc.30857

[CR61] Willett W (1998) Recall of remote diet. Willett W. Nutritional epidemiology. Oxford University Press, New York, pp 148–156

[CR62] Zablotska LB, Ron E, Rozhko AV, Hatch M, Polyanskaya ON, Brenner AV, Lubin J, Romanov GN, McConnell RJ, O’Kane P, Evseenko VV, Drozdovitch VV, Luckyanov N, Minenko VF, Bouville A, Masyakin VB (2011) Thyroid cancer risk in Belarus among children and adolescents exposed to radioiodine after the chornobyl accident. Br J Cancer 104:181–187. 10.1038/sj.bjc.6605967 21102590 10.1038/sj.bjc.6605967 PMC3039791

[CR63] Zhumadilov K, Ivannikov A, Apsalikov KN, Zhumadilov Z, Toyoda S, Zharlyganova D, Tieliewuhan E, Endo S, Tanaka K, Miyazawa C, Okamoto T, Hoshi M (2006) Radiation dose Estimation by tooth enamel EPR dosimetry for residents of Dolon and Bodene. J Radiat Res 47(Suppl A). 10.1269/jrr.47.a47. ):A47 – 53 10.1269/jrr.47.a47 16571944

[CR65] Zhumadilov K, Ivannikov A, Stepanenko V, Zharlyganova D, Toyoda S, Zhumadilov Z, Hoshi M (2013) ESR dosimetry study of population in the vicinity of the Semipalatinsk nuclear test site. J Radiat Res 54:775–779. 10.1093/jrr/rrt008 23404205 10.1093/jrr/rrt008 PMC3709679

[CR66] Zvonova IA (1989) Dietary intake of stable I and some aspects of radioiodine dosimetry. Health Phys 57:471–475 2777553

